# The Complex Neutrosophic Soft Expert Relation and Its Multiple Attribute Decision-Making Method

**DOI:** 10.3390/e20020101

**Published:** 2018-01-31

**Authors:** Ashraf Al-Quran, Nasruddin Hassan

**Affiliations:** School of Mathematical Sciences, Faculty of Science and Technology, Universiti Kebangsaan Malaysia, Bangi 43600, Selangor, Malaysia

**Keywords:** complex neutrosophic set, complex neutrosophic soft expert set, decision-making, single-valued neutrosophic set, neutrosophic soft relation

## Abstract

This paper introduces a novel soft computing technique, called the complex neutrosophic soft expert relation (CNSER), to evaluate the degree of interaction between two hybrid models called complex neutrosophic soft expert sets (CNSESs). CNSESs are used to represent two-dimensional data that are imprecise, uncertain, incomplete and indeterminate. Moreover, it has a mechanism to incorporate the parameter set and the opinions of all experts in one model, thus making it highly suitable for use in decision-making problems where the time factor plays a key role in determining the final decision. The complex neutrosophic soft expert set and complex neutrosophic soft expert relation are both defined. Utilizing the properties of CNSER introduced, an empirical study is conducted on the relationship between the variability of the currency exchange rate and Malaysian exports and the time frame (phase) of the interaction between these two variables. This study is supported further by an algorithm to determine the type and the degree of this relationship. A comparison between different existing relations and CNSER to show the ascendancy of our proposed CNSER is provided. Then, the notion of the inverse, complement and composition of CNSERs along with some related theorems and properties are introduced. Finally, we define the symmetry, transitivity and reflexivity of CNSERs, as well as the equivalence relation and equivalence classes on CNSESs. Some interesting properties are also obtained.

## 1. Introduction

Zadeh [1] introduced the concept of the fuzzy set to represent uncertain data, which is abounding in the real world, and it was extended by Atanassov [2] to the intuitionistic fuzzy set. These sets have been applied to vague soft sets [3,4,5] and many real-life problems in uncertain and ambiguous environment [6,7,8]. The words neutrosophy and neutrosophic were coined by Smarandache [9], who later defined neutrosophic sets [10] as natural extensions of fuzzy and intuitionistic fuzzy sets. In order to apply the neutrosophic set to real-life problems, its operators need to be specified. Thus, the basic operations of the single-valued neutrosophic set (SVNS) were defined [11]. Subsequently, the single-valued neutrosophic set was applied extensively in the multiple attribute decision-making (MADM) methods of operational research such as TOPSIS [12], COPRAS [13], VIKOR [14], WASPAS [15], MULTIMOORA [16], EDAS [17], TODIM [18] and others. Since Molodtsov [19] proposed the soft set, hybrids of fuzzy and soft sets have been developed such as the fuzzy soft set [20], intuitionistic fuzzy soft set [21], neutrosophic soft set [22], soft expert set [23], intuitionistic fuzzy soft expert set [24] and single-valued neutrosophic soft expert set (SVNSES) [25].

Fuzzy set theory was then extended from the real to the complex field by Ramot et al. [26], which progressed rapidly to the complex fuzzy logic [27] and complex intuitionistic fuzzy sets (CIFS) [28]. Nevertheless, these sets do not have structures to handle imprecise, inconsistent, indeterminate and incomplete data that are periodic in nature. Therefore, Ali and Smarandache [29] introduced the complex neutrosophic set. In complex neutrosophic sets, each membership function is associated with a phase term. This feature gives wave-like properties that could be used to describe constructive and destructive interference depending on the phase value of an element, as well as its ability to deal with indeterminacy.

However, the complex neutrosophic set lacks the adequate parameterization tool to facilitate the representation of parameters, and it it does not have a mechanism to incorporate the opinion of all experts in one model. Thus, the complex neutrosophic soft expert set (CNSES) is proposed to provide a more adequate parameterization tool that can represent the problem parameters in a more comprehensive and complete manner. It has also the added advantage of allowing the users to know the opinion of all the experts in a single model without the need for any additional cumbersome operations. This increases the validity of this model as most real-life situations deal with elements and parameters that are subjective and biased and have the potential to be distorted and interpreted differently by different parties.

In this paper, we introduce and study the various types of relations between CNSESs. A great deal of researchers have studied the relations between fuzzy sets [30,31], fuzzy soft sets and their generalizations [32,33,34]. However, the most significant shortcoming of these relations is their inability to capture information pertaining to the time frame of the interaction between the parameters, which is a very important component of most real-life situations. Motivated by these studies, we herein introduce the complex neutrosophic soft expert relation (CNSER) for which the ranges of its membership functions are represented in terms of complex numbers, which give it the ability to represent the degree and the phase of the interaction between the elements of the CNSESs. CNSER is equipped with adequate parameterization and created with the opinions of all experts. It also has the ability to handle the imprecise, indeterminate, inconsistent and incomplete information that is captured by the amplitude terms and phase terms of the complex numbers, simultaneously. A novel approach to MADM problems based on CNSES is also introduced. This approach converts the CNSES to a SVNSES using a practical and useful algorithm, which highlights the role of the time factor in determining the final decision. The advantages of our models and the drawbacks of the current methods discussed above served as the motivation for this paper.

There are four main contributions of this paper. Firstly, we introduce the concept of CNSES, which combines the advantages of both the complex neutrosophic set and soft expert set. Secondly, we define the concept of the Cartesian product of two CNSESs and then study the relation between these two sets by establishing the concept of CNSERs. Thirdly, we use the CNSER together with a generalized algorithm to determine the degree and the type of the influence of volatility of exchange rates on the total exports of Malaysia and then deduce results that help to make more accurate economical decisions in the future. This study is supported by a comparison among the proposed method and other methods to reveal the superiority of our proposed method. Lastly, the concepts of inverse, complement, composition, equivalence relation and equivalence classes were extended to the notion of CNSER.

The organization of this paper is as follows. Section 2 provides a brief synopsis of the pertinent literature where we review some background on neutrosophic and complex neutrosophic set. The CNSES is also introduced in this section along with an illustrative example. Throughout Section 3, we define the concept of the Cartesian product of two CNSESs. Subsequently, the concept of CNSERs will be defined. Then, the CNSER is used to solve a MADM problem in the economy. We end this section by making a comparison among CNSER and other existing models. In Section 4, we present some operations on CNSER along with some theorems and propositions. Section 5, demonstrates the equivalence relation and equivalence classes on CNSESs. In the final section, the conclusions of the findings of the paper are stated. This section also provides suggestions for further research in CNSERs.

## 2. Preliminaries

In this section, we recapitulate the concepts of neutrosophic and complex neutrosophic sets and present an overview of the operation structures of the complex neutrosophic model that are relevant to the work in this paper. The CNSES is also introduced.

**Definition** **1** ([10])**.** *Let U be a universe of discourse. A neutrosophic set N in U is defined as: $A=\{<u;T_{N}\left(u\right);I_{N}\left(u\right);F_{N}\left(u\right)>;u\in U\}$ where $T_{N}\left(u\right),I_{N}\left(u\right)$ and $F_{N}\left(u\right)$ are the truth membership function, the indeterminacy membership function and the falsity membership function, respectively, such that T; I; ${F:X\to ]}^{-}0;1^{+}[$ and ${}^{-}0\leq T_{N}\left(u\right)+I_{N}\left(u\right)+F_{N}\left(u\right)\leq 3^{+}.$*

In order to apply the neutrosophic set to scientific fields, its parameters should be specified. Hence, Wang et al. [11] provided the following definition.

**Definition** **2** ([11])**.** *Let U be a universe of discourse. A single-valued neutrosophic set (SVNS) S in U is defined as:*

$$S=\int_{U}\langle T\left(U\right),I\left(U\right),F\left(U\right)\rangle /u,\;u\in U,$$

*when U is continuous and*

$$S=\sum_{i=1}^{n}\langle T\left(U_{i}\right),I\left(U_{i}\right),F\left(U_{i}\right)\rangle /u_{i},\;u_{i}\in U,$$

*when U is discrete, where $T_{S}$, $I_{S}$ and $F_{S}$ are the truth membership function, the indeterminacy membership function and the falsity membership function, respectively, and $T_{S}$; $I_{S}$; $F_{S}$ : U→[0,1].*

Ali and Smarandache [29] conceptualized the complex neutrosophic set and gave the basic operations in the following two definitions.

**Definition** **3** ([29])**.** *Let a universe of discourse U, a complex neutrosophic set S in U, be characterized by a truth membership function $T_{S}\left(u\right)$, an indeterminacy membership function $I_{S}\left(u\right)$ and a falsity membership function $F_{S}\left(u\right)$ that assigns a complex-valued grade for each of these membership functions in S for any u∈U. By definition, the values $T_{S}\left(u\right)$, $I_{S}\left(u\right)$, $F_{S}\left(u\right)$ and their sum may all be within the unit circle in the complex plane and are of the form, $T_{S}\left(u\right)=p_{S}\left(u\right).e^{j\mu_{S}\left(u\right)}$, $I_{S}\left(u\right)=q_{S}\left(u\right).e^{j\nu_{S}\left(u\right)}$ and $F_{S}\left(u\right)=r_{S}\left(u\right).e^{j\omega_{S}\left(u\right)}$; each of $p_{S}\left(u\right),q_{S}\left(u\right),r_{S}\left(u\right)$ and $\mu_{S}\left(u\right),\nu_{S}\left(u\right),\omega_{S}\left(u\right)$ are, respectively, real valued and $p_{S}\left(u\right),q_{S}\left(u\right),r_{S}\left(u\right)\in \left[0,1\right]$ such that $0^{-}\leq P_{S}\left(u\right)+q_{S}\left(u\right)+r_{S}\left(u\right)\leq 3^{+}.$*

**Definition** **4** ([29])**.** *Let A and B be two complex neutrosophic sets on the universe U, where A is characterized by a truth membership function $T_{A}\left(u\right)=p_{A}\left(u\right).e^{j\mu_{A}\left(u\right)}$, an indeterminacy membership function $I_{A}\left(u\right)=q_{A}\left(u\right).e^{j\nu_{A}\left(u\right)}$ and a falsity membership function $F_{A}\left(u\right)=r_{A}\left(u\right).e^{j\omega_{A}\left(u\right)}$ and B is characterized by a truth membership function $T_{B}\left(u\right)=p_{B}\left(u\right).e^{j\mu_{B}\left(u\right)}$, an indeterminacy membership function $I_{B}\left(u\right)=q_{B}\left(u\right).e^{j\nu_{B}\left(u\right)}$ and a falsity membership function $F_{B}\left(u\right)=r_{B}\left(u\right).e^{j\omega_{B}\left(u\right)}$.*

*We define the the complement, subset, union and intersection operations as follows.*

*1.*  The complement of A, denoted as $\tilde{c}\left(A\right)$, is specified by functions:
  $T_{\tilde{c}\left(A\right)}\left(u\right)=p_{\tilde{c}\left(A\right)}\left(u\right).e^{j\mu_{\tilde{c}\left(A\right)}\left(u\right)}=r_{A}\left(u\right).e^{j(2\pi -\mu_{A}\left(u\right))}$,
  $I_{\tilde{c}\left(A\right)}\left(u\right)=q_{\tilde{c}\left(A\right)}\left(u\right).e^{j\nu_{\tilde{c}\left(A\right)}\left(u\right)}=\left(1-q_{A}\left(u\right)\right).e^{j(2\pi -\nu_{A}\left(u\right))}$ and
  $F_{\tilde{c}\left(A\right)}\left(u\right)=r_{\tilde{c}\left(A\right)}\left(u\right).e^{j\omega_{\tilde{c}\left(A\right)}\left(u\right)}=p_{A}\left(u\right).e^{j(2\pi -\omega_{A}\left(u\right))}$.
*2.*  *A is said to be complex neutrosophic subset of B (A⊆B) if and only if the following conditions are satisfied:*
  *(a)*  $T_{A}\left(u\right)\leq T_{B}\left(u\right)$ such that $p_{A}\left(u\right)\leq p_{B}\left(u\right)$ and $\mu_{A}\left(u\right)\leq \mu_{B}\left(u\right)$.
  *(b)*  $I_{A}\left(u\right)\geq I_{B}\left(u\right)$ such that $q_{A}\left(u\right)\geq q_{B}\left(u\right)$ and $\nu_{A}\left(u\right)\geq \nu_{B}\left(u\right)$.
  *(c)*  $F_{A}\left(u\right)\geq F_{B}\left(u\right)$ such that $r_{A}\left(u\right)\geq r_{B}\left(u\right)$ and $\omega_{A}\left(u\right)\geq \omega_{B}\left(u\right)$.
*3.*  The union (intersection) of A and B, denoted as A∪(∩)B, and the truth membership function $T_{A\cup (\cap )B}\left(u\right)$, the indeterminacy membership function $I_{A\cup (\cap )B}\left(u\right)$ and the falsity membership function $F_{A\cup (\cap )B}\left(u\right)$ are defined as:
  $T_{A\cup (\cap )B}\left(u\right)=\left[\left(p_{A}\left(u\right)\vee \left(\wedge \right)p_{B}\left(u\right)\right)\right].e^{j(\mu_{A}\left(u\right)\vee \left(\wedge \right)\mu_{B}\left(u\right))},$
  $I_{A\cup (\cap )B}\left(u\right)=\left[\left(q_{A}\left(u\right)\wedge \left(\vee \right)q_{B}\left(u\right)\right)\right].e^{j(\nu_{A}\left(u\right)\wedge \left(\vee \right)\nu_{B}\left(u\right))}$ and
  $F_{A\cup (\cap )B}\left(u\right)=\left[\left(r_{A}\left(u\right)\wedge \left(\vee \right)r_{B}\left(u\right)\right)\right].e^{j(\omega_{A}\left(u\right)\wedge \left(\vee \right)\omega_{B}\left(u\right))}$,
  where ∨ = max and ∧ = min.

We will now introduce the concept of CNSES.

**Definition** **5.** *Let U be a universe, E a set of parameters, X a set of experts (agents) and O={1=agree,0=disagree} a set of opinions. Let Z=E×X×O and A⊆Z. A pair (H,A) is called a complex neutrosophic soft expert set (CNSES) over U, where H is a mapping given by:*

$$H:A\to CN^{U},$$

*where $CN^{U}$ denotes the power complex neutrosophic set of U.*

It is to be noted that ∀α∈A, H(α) represents the degree and the phase of belongingness, indeterminacy and non-belongingness of the elements of U in H(α).

The following example illustrates the above definition of the CNSES.

**Example** **1.** *Suppose we want to examine the influence of some financial indicators on the most important sectors in the Malaysian economy, which are represented by the universal set $U=\left\{u_{1},u_{2}\right\}$, where $u_{1}$ stands for the industry sector and $u_{2}$ stands for the tourism sector. Let $E=\left\{e_{1},e_{2},e_{3}\right\}$ be the set of parameters (financial indicators), where $e_{i}\left(i=1,2,3\right)$ denotes the indicators “the plunge in oil prices”, “currency fluctuations” and “the rate of inflation”. Let $X=\left\{p,q\right\}$ be a set of experts who are assigned to analyze these three indicators by determining the degree and the total time of the influence of these factors on the mentioned sectors of the Malaysian economy. Suppose O={1=agree,0=disagree} is a set of opinions and Z=E×X×O, where A⊆Z. Suppose that this study is conducted over a limited time span of 12 months. The experts can express their opinions as exemplified below.*

$$\begin{array}{cc} (H,A)= & \{\left\{\left(e_{1},p,1\right),\left\{\frac{\langle 0.9e^{j2\Pi (11/12)},\;0.2e^{j2\Pi (3/12)},\;0.1e^{j2\Pi (0)}\rangle }{u_{1}},\frac{\langle 0.9e^{j2\Pi (11/12)},\;0.6e^{j2\Pi (5/12)},\;0.3e^{j2\Pi (0)}\rangle }{u_{2}}\right\}\right\},\\ & \left\{\left(e_{1},q,1\right),\left\{\frac{\langle 0.5e^{j2\Pi (3/24)},\;0.2e^{j2\Pi (1/48)},\;0.9e^{j2\Pi (10/12)}\rangle }{u_{1}},\frac{\langle 0.9e^{j2\Pi (9/12)},\;0.4e^{j2\Pi (5/12)},\;0.5e^{j2\Pi (5/12)}\rangle }{u_{2}}\right\}\right\},\\ & \left\{\left(e_{2},p,1\right),\left\{\frac{\langle 0.3e^{j2\Pi (6/12)},\;0.2e^{j2\Pi (1/24)},\;0.9e^{j2\Pi (47/48)}\rangle }{u_{1}},\frac{\langle 0.9e^{j2\Pi (11/12)},\;0.5e^{j2\Pi (7/12)},\;0.4e^{j2\Pi (1/48)}\rangle }{u_{2}}\right\}\right\},\\ & \left\{\left(e_{2},q,1\right),\left\{\frac{\langle 0.1e^{j2\Pi (1/24)},\;0.2e^{j2\Pi (5/12)},\;0.9e^{j2\Pi (11/12)}\rangle }{u_{1}},\frac{\langle 0.9e^{j2\Pi (9/12)},\;0.1e^{j2\Pi (7/12)},\;0.2e^{j2\Pi (0)}\rangle }{u_{2}}\right\}\right\},\\ & \left\{\left(e_{3},p,1\right),\left\{\frac{\langle 0.5e^{j2\Pi (9/12)},\;0.3e^{j2\Pi (1/48)},\;0.8e^{j2\Pi (10/12)}\rangle }{u_{1}},\frac{\langle 0.8e^{j2\Pi (5/12)},\;0.2e^{j2\Pi (3/12)},\;0.1e^{j2\Pi (0)}\rangle }{u_{2}}\right\}\right\},\\ & \left\{\left(e_{3},q,1\right),\left\{\frac{\langle 0.6e^{j2\Pi (8/12)},\;0.3e^{j2\Pi (9/12)},\;0.7e^{j2\Pi (11/12)}\rangle }{u_{1}},\frac{\langle 0.8e^{j2\Pi (3/12)},\;0.1e^{j2\Pi (0.1)},\;0.3e^{j2\Pi (5/24)}\rangle }{u_{2}}\right\}\right\},\\ & \left\{\left(e_{1},p,0\right),\left\{\frac{\langle 0.1e^{j2\Pi (8/12)},\;0.8e^{j2\Pi (7/12)},\;0.7e^{j2\Pi (10/12)}\rangle }{u_{1}},\frac{\langle 0.3e^{j2\Pi (5/12)},\;0.4e^{j2\Pi (8/12)},\;0.9e^{j2\Pi (1)}\rangle }{u_{2}}\right\}\right\},\\ & \left\{\left(e_{1},q,0\right),\left\{\frac{\langle 0.9e^{j2\Pi (11/12)},\;0.8e^{j2\Pi (1/12)},\;0.5e^{j2\Pi (1/24)}\rangle }{u_{1}},\frac{\langle 0.5e^{j2\Pi (5/12)},\;0.6e^{j2\Pi (8/12)},\;0.9e^{j2\Pi (5/12)}\rangle }{u_{2}}\right\}\right\},\\ & \left\{\left(e_{2},p,0\right),\left\{\frac{\langle 0.9e^{j2\Pi (1/24)},\;0.8e^{j2\Pi (3/12)},\;0.3e^{j2\Pi (9/12)}\rangle }{u_{1}},\frac{\langle 0.4e^{j2\Pi (7/12)},\;0.1e^{j2\Pi (5/12)},\;0.4e^{j2\Pi (7/12)}\rangle }{u_{2}}\right\}\right\},\\ & \left\{\left(e_{2},q,0\right),\left\{\frac{\langle 0.9e^{j2\Pi (9/12)},\;0.8e^{j2\Pi (10/12)},\;0.1e^{j2\Pi (1/12)}\rangle }{u_{1}},\frac{\langle 0.2e^{j2\Pi (1/12)},\;0.9e^{j2\Pi (47/48)},\;0.2e^{j2\Pi (1)}\rangle }{u_{2}}\right\}\right\},\\ & \left\{\left(e_{3},p,0\right),\left\{\frac{\langle 0.8e^{j2\Pi (10/12)},\;0.8e^{j2\Pi (9/12)},\;0.1e^{j2\Pi (1/24)}\rangle }{u_{1}},\frac{\langle 0.2e^{j2\Pi (3/12)},\;0.9e^{j2\Pi (11/12)},\;0.2e^{j2\Pi (5/12)}\rangle }{u_{2}}\right\}\right\},\\ & \left\{\left(e_{3},q,0\right),\left\{\frac{\langle 0.9e^{j2\Pi (11/12)},\;0.8e^{j2\Pi (10/12)},\;0.1e^{j2\Pi (3/12)}\rangle }{u_{1}},\frac{\langle 0.2e^{j2\Pi (4/12)},\;0.9e^{j2\Pi (11/12)},\;0.2e^{j2\Pi (1)}\rangle }{u_{2}}\right\}\right\}\} \end{array}$$

In the CNSES (H,A), the amplitude terms represent the degree of influence of a financial indicator on the corresponding economy sector, whereas the phase terms depict the total time (phase) of this influence. Both the amplitude terms and phase terms lie between zero and one such that an amplitude term with a value close to zero (one) implies that a financial indicator has very little (strong) influence on its corresponding economy sector, and a phase term with a value close to zero (one) implies that this influence spans for a very long (short) period of time.

## 3. Complex Neutrosophic Soft Expert Relations

We will propose the definitions of Cartesian product of CNSESs and the complex neutrosophic soft expert relation.

We define the Cartesian product between two CNSESs as follows:

**Definition** **6.** *Let U be an initial universal set, E a set of parameters, X a set of experts and O={1=agree,0=disagree} a set of opinions. Let Z=E×X×O and A,B⊆Z. Let (H,A) and (G,B) be two complex neutrosophic soft expert sets over the soft universe (U,Z). The Cartesian product of (H,A) and (G,B), denoted by (H,A)×(G,B), is a complex neutrosophic soft expert set (K,C), where C=A×B and (K,C) are defined as $\left(K,C\right)=\left\{\langle \left(a,b\right),T_{K(a,b)}\left(u\right),I_{K(a,b)}\left(u\right),F_{K(a,b)}\left(u\right)\rangle :\left(a,b\right)\in A\times B,u\in U\right\},$ where $T_{K(a,b)}\left(u\right)$ is a complex-valued membership function, $I_{K(a,b)}\left(u\right)$ is a complex-valued indeterminacy membership function, $F_{K(a,b)}\left(u\right)$ is a complex-valued non-membership function and ∀u∈U, ∀(a,b)∈A×B,*

$$\begin{array}{cc} T_{K(a,b)}\left(u\right) & =p_{K(a,b)}\left(u\right).e^{j\mu_{K(a,b)}\left(u\right)}=min\left(p_{H(a)}\left(u\right),p_{G(b)}\left(u\right)\right).e^{jmin(\mu_{H(a)}\left(u\right),\mu_{G(b)}\left(u\right))},\\ I_{K(a,b)}\left(u\right) & =q_{K(a,b)}\left(u\right).\;e^{j\nu_{K(a,b)}\left(u\right)}=max\left(q_{H(a)}\left(u\right),q_{G(b)}\left(u\right)\right).\;e^{jmax(\nu_{H(a)}\left(u\right),\nu_{G(b)}\left(u\right))}\;and\\ F_{K(a,b)}\left(u\right) & =r_{K(a,b)}\left(u\right).\;e^{j\omega_{K(a,b)}\left(u\right)}=max\left(r_{H(a)}\left(u\right),r_{G(b)}\left(u\right)\right).\;e^{jmax(\omega_{H(a)}\left(u\right),\omega_{G(b)}\left(u\right))}. \end{array}$$

We will now define the concept of the complex neutrosophic soft expert relation as follows.

**Definition** **7.** Let (H,A) and (G,B) be two CNSESs over a universe U. A complex neutrosophic soft expert relation (CNSER) from (H,A) to (G,B) is a complex neutrosophic soft expert subset of (H,A)×(G,B). Thus, a complex neutrosophic soft expert relation from (H,A) to (G,B) is of the form (R,C) where C⊆A×B and R(a,b)⊆H(a)×G(b), ∀(a,b)∈C.

*The CNSER (R,C) from (H,A) to (G,B) can be written as $\left(R,C\right)=H\left(a\right){\tilde{\cap }}_{N}G\left(b\right),\forall \left(a,b\right)\in C\subseteq A\times B.$ As always, (R,C) can be represented as the set of ordered pairs:*

$$\left(R,C\right)=\left\{\langle \left(a,b\right),T_{R(a,b)}\left(u\right),I_{R(a,b)}\left(u\right),F_{R(a,b)}\left(u\right)\rangle :\left(a,b\right)\in C\subseteq A\times B,u\in U\right\},$$

*where ∀u∈U and ∀(a,b)∈C⊆A×B,*

$$\begin{array}{cc} T_{R(a,b)}\left(u\right) & =p_{R(a,b)}\left(u\right).e^{j\mu_{R(a,b)}\left(u\right)}=min\left(p_{H(a)}\left(u\right),p_{G(b)}\left(u\right)\right).e^{jmin(\mu_{H(a)}\left(u\right),\mu_{G(b)}\left(u\right))},\\ I_{R(a,b)}\left(u\right) & =q_{R(a,b)}\left(u\right).\;e^{j\nu_{R(a,b)}\left(u\right)}=max\left(q_{H(a)}\left(u\right),q_{G(b)}\left(u\right)\right).\;e^{jmax(\nu_{H(a)}\left(u\right),\nu_{G(b)}\left(u\right))}\;and\\ F_{R(a,b)}\left(u\right) & =r_{R(a,b)}\left(u\right).e^{j\omega_{R(a,b)}\left(u\right)}=max\left(r_{H(a)}\left(u\right),r_{G(b)}\left(u\right)\right).\;e^{jmax(\omega_{H(a)}\left(u\right),\omega_{G(b)}\left(u\right))}. \end{array}$$

*If (R,C) is a CNSER from (H,A) to (H,A), then it is called a CNSER on (H,A), and it can be defined in the parameterized form as follows.*

$$If\left(H,A\right)=\left\{H\left(a_{1}\right),H\left(a_{2}\right),\ldots \right\},\;then\;H\left(a_{1}\right)RH\left(a_{2}\right)if\;and\;only\;if\;H\left(a_{1}\right)\times H\left(a_{2}\right)\in \left(R,C\right).$$

Now, we put forward a real-life application of the CNSER to reveal its ability to describe and analyze real events.

### 3.1. Complex Neutrosophic Soft Expert Relation in the Economy

The complex neutrosophic soft expert set relation can be effectively used to measure the interaction between several economical variables where the factor of time plays a key role and the indeterminacy is unavoidable.

Now, we give an example of a relation between two CNSESs.

**Example** **2.** Suppose that we are curious about the changes of the monetary rate of exchange between the Malaysian ringgit (MYR) and the currencies of its four major trading partners; U.S. dollar (USD), Chinese yuan (yuan), Japanese yen (JPY) and Singapore dollar (SD) based on exports of Malaysia to these countries within a time frame of 12 months. Let U be the universal set, which describes the degree and type of influence of the parameters (financial indicators) related to the Malaysian economy, where $U=\{u_{1},u_{2},u_{3}\}$ such that $u_{1}=$ positive influence, $u_{2}=$ negative influence and $u_{3}=$ no influence. Let $E=\{e_{1},e_{2},e_{3},e_{4},{\overset{´}{e}}_{1},{\overset{´}{e}}_{2},{\overset{´}{e}}_{3},{\overset{´}{e}}_{4}\}$ be a set of parameters that represents the financial indicators that affect the Malaysian economy, where $e_{1},e_{2},e_{3}$ and $e_{4}$ = volatility of currency rates between MYR and USD, yuan, JPY and SD, respectively, and ${\overset{´}{e}}_{1},{\overset{´}{e}}_{2},{\overset{´}{e}}_{3},{\overset{´}{e}}_{4}$ = total exports of Malaysia to the U.S., China, Japan and Singapore, respectively. Let X={p,q} be a set of economic experts who are assigned to analyze the effect of the volatility of exchange rates on the total exports of Malaysia by determining the degree and the total time of this influence on the total exports of Malaysia. Let (H,A) and (G,B) be two CNSESs over U, where $A=\{\left(e_{1},p,1\right),\left(e_{1},q,1\right),\left(e_{1},p,0\right),\left(e_{1},q,0\right),\left(e_{2},p,1\right),\left(e_{2},q,1\right),\left(e_{2},p,0\right),\left(e_{2},q,0\right)$, $\left(e_{3},p,1\right),\left(e_{3},q,1\right),\left(e_{3},p,0\right),\left(e_{3},q,0\right),\left(e_{4},p,1\right),\left(e_{4},q,1\right),\left(e_{4},p,0\right),\left(e_{4},q,0\right)\}$ and $B=\{\left({\overset{´}{e}}_{1},p,1\right),\left({\overset{´}{e}}_{1},q,1\right)$, $\left({\overset{´}{e}}_{1},p,0\right),\left({\overset{´}{e}}_{1},q,0\right),\left({\overset{´}{e}}_{2},p,1\right),\left({\overset{´}{e}}_{2},q,1\right),\left({\overset{´}{e}}_{2},p,0\right)$, $\left({\overset{´}{e}}_{2},q,0\right),\left({\overset{´}{e}}_{3},p,1\right),\left({\overset{´}{e}}_{3},q,1\right),\left({\overset{´}{e}}_{3},p,0\right),\left({\overset{´}{e}}_{3},q,0\right),\left({\overset{´}{e}}_{4},p,1\right)$, $\left({\overset{´}{e}}_{4},q,1\right),\left({\overset{´}{e}}_{4},p,0\right),\left({\overset{´}{e}}_{4},q,0\right)\}$.

*Suppose the two CNSESs (H,A) and (G,B) are defined as follows.*

$$\begin{array}{cc} (H,A)= & \{(\left(e_{1},p,1\right),\{\frac{\langle 0.01e^{j2\Pi (1/48)},\;0.2e^{j2\Pi (1/24)},\;0.9e^{j2\Pi (12/12)}\rangle }{u_{1}},\frac{\langle 0.9e^{j2\Pi (11/12)},\;0.1e^{j2\Pi (4/12)},\;0.05e^{j2\Pi (0)}\rangle }{u_{2}},\\ & \frac{\langle 0.04e^{j2\Pi (0)},\;0.2e^{j2\Pi (1/48)},\;0.7e^{j2\Pi (11/12)}\rangle }{u_{3}}\}),(\left(e_{1},q,1\right),\{\frac{\langle 0.02e^{j2\Pi (0)},\;0.1e^{j\Pi (1/24)},\;0.8e^{j2\Pi (11/12)}\rangle }{u_{1}},\\ & \frac{\langle 0.9e^{j2\Pi (10/12)},\;0.1e^{j2\Pi (3/12)},\;0.07e^{j2\Pi (1/48)}\rangle }{u_{2}},\frac{\langle 0.05e^{j2\Pi (1/24)},\;0.4e^{j2\Pi (1/12)},\;0.6e^{j2\Pi (10/12)}\rangle }{u_{3}}\}),(\left(e_{1},p,0\right),\\ & \{\frac{\langle 0.9e^{j2\Pi (47/48)},\;0.8e^{j2\Pi (23/24)},\;0.01e^{j2\Pi (0)}\rangle }{u_{1}},\frac{\langle 0.05e^{j2\Pi (1/12)},\;0.9e^{j2\Pi (8/12)},\;0.9e^{j2\Pi (12/12)}\rangle }{u_{2}},\\ & \frac{\langle 0.7e^{j2\Pi (12/12)},\;0.8e^{j2\Pi (47/48)},\;0.04e^{j2\Pi (1/12)}\rangle }{u_{3}}\}),(\left(e_{1},q,0\right),\{\frac{\langle 0.8e^{j2\Pi (12/12)},\;0.9e^{j\Pi (23/24)},\;0.02e^{j2\Pi (1/12)}\rangle }{u_{1}},\\ & \frac{\langle 0.07e^{j2\Pi (2/12)},\;0.9e^{j2\Pi (9/12)},\;0.9e^{j2\Pi (47/48)}\rangle }{u_{2}},\frac{\langle 0.6e^{j2\Pi (23/24)},\;0.6e^{j2\Pi (11/12)},\;0.05e^{j2\Pi (2/12)}\rangle }{u_{3}}\}),(\left(e_{2},p,1\right),\\ & \{\frac{\langle 0.05e^{j2\Pi (0)},\;0.1e^{j2\Pi (1/48)},\;0.7e^{j2\Pi (10/12)}\rangle }{u_{1}},\frac{\langle 0.9e^{j2\Pi (9/12)},\;0.3e^{j2\Pi (1/12)},\;0.08e^{j2\Pi (1/48)}\rangle }{u_{2}},\\ & \frac{\langle 0.03e^{j2\Pi (1/24)},\;0.4e^{j2\Pi (0)},\;0.9e^{j2\Pi (11/12)}\rangle }{u_{3}}\}),(\left(e_{2},q,1\right),\{\frac{\langle 0.02e^{j2\Pi (1/24)},\;0.2e^{j2\Pi (1/24)},\;0.8e^{j2\Pi (12/12)}\rangle }{u_{1}},\\ & \frac{\langle 0.8e^{j2\Pi (10/12)},\;0.3e^{j2\Pi (2/12)},\;0.08e^{j2\Pi (1/48)}\rangle }{u_{2}},\frac{\langle 0.06e^{j2\Pi (0)},\;0.1e^{j2\Pi (1/48)},\;0.9e^{j2\Pi (11/12)}\rangle }{u_{3}}\}),(\left(e_{2},p,0\right),\\ & \{\frac{\langle 0.7e^{j2\Pi (12/12)},\;0.9e^{j2\Pi (47/48)},\;0.05e^{j2\Pi (2/12)}\rangle }{u_{1}},\frac{\langle 0.08e^{j2\Pi (3/12)},\;0.7e^{j2\Pi (11/12)},\;0.9e^{j2\Pi (47/48)}\rangle }{u_{2}},\\ & \frac{\langle 0.9e^{j2\Pi (23/24)},\;0.6e^{j2\Pi (12/12)},\;0.03e^{j2\Pi (1/12)}\rangle }{u_{3}}\}),(\left(e_{2},q,0\right),\{\frac{\langle 0.8e^{j2\Pi (23/24)},\;0.8e^{j2\Pi (23/24)},\;0.02e^{j2\Pi (0)}\rangle }{u_{1}},\\ & \frac{\langle 0.08e^{j2\Pi (2/12)},\;0.7e^{j2\Pi (10/12)},\;0.8e^{j2\Pi (47/48)}\rangle }{u_{2}},\frac{\langle 0.9e^{j2\Pi (12/12)},\;0.9e^{j2\Pi (47/48)},\;0.06e^{j2\Pi (1/12)}\rangle }{u_{3}}\}),(\left(e_{3},p,1\right),\\ & \{\frac{\langle 0.9e^{j2\Pi (11/12)},\;0.1e^{j\Pi (0)},\;0.02e^{j2\Pi (0)}\rangle }{u_{1}},\frac{\langle 0.01e^{j2\Pi (0)},\;0.1e^{j2\Pi (1/24)},\;0.7e^{j2\Pi (9/12)}\rangle }{u_{2}},\\ & \frac{\langle 0.05e^{j2\Pi (1/12)},\;0.4e^{j2\Pi (2/12)},\;0.6e^{j2\Pi (10/12)}\rangle }{u_{3}}\}),(\left(e_{3},q,1\right),\{\frac{\langle 0.8e^{j2\Pi (10/12)},\;0.2e^{j\Pi (1/48)},\;0.03e^{j2\Pi (0)}\rangle }{u_{1}},\\ & \frac{\langle 0.04e^{j2\Pi (0)},\;0.2e^{j2\Pi (3/24)},\;0.6e^{j2\Pi (10/12)}\rangle }{u_{2}},\frac{\langle 0.02e^{j2\Pi (3/12)},\;0.5e^{j2\Pi (1/12)},\;0.5e^{j2\Pi (11/12)}\rangle }{u_{3}}\}),(\left(e_{3},p,0\right),\\ & \{\frac{\langle 0.02e^{j2\Pi (1/12)},\;0.9e^{j\Pi (1)},\;0.9e^{j2\Pi (1)}\rangle }{u_{1}},\frac{\langle 0.7e^{j2\Pi (1)},\;0.9e^{j2\Pi (23/24)},\;0.01e^{j2\Pi (3/12)}\rangle }{u_{2}},\\ & \frac{\langle 0.6e^{j2\Pi (11/12)},\;0.6e^{j2\Pi (10/12)},\;0.05e^{j2\Pi (2/12)}\rangle }{u_{3}}\}),(\left(e_{3},q,0\right),\{\frac{\langle 0.03e^{j2\Pi (2/12)},\;0.8e^{j\Pi (47/48)},\;0.8e^{j2\Pi (1)}\rangle }{u_{1}},\\ & \frac{\langle 0.6e^{j2\Pi (1)},\;0.2e^{j2\Pi (21/24)},\;0.04e^{j2\Pi (2/12)}\rangle }{u_{2}},\frac{\langle 0.5e^{j2\Pi (9/12)},\;0.5e^{j2\Pi (11/12)},\;0.02e^{j2\Pi (1/12)}\rangle }{u_{3}}\}),(\left(e_{4},p,1\right),\\ & \{\frac{\langle 0.1e^{j2\Pi (1/12)},\;0.5e^{j2\Pi (4/12)},\;0.9e^{j2\Pi (12/12)}\rangle }{u_{1}},\frac{\langle 0.06e^{j2\Pi (0)},\;0.5e^{j2\Pi (7/12)},\;0.8e^{j2\Pi (11/12)}\rangle }{u_{2}},\\ & \frac{\langle 1e^{j2\Pi (12/12)},\;0.1e^{j2\Pi (1/12)},\;0.09e^{j2\Pi (1/48)}\rangle }{u_{3}}\}),(\left(e_{4},q,1\right),\{\frac{\langle 0.2e^{j2\Pi (3/12)},\;0.3e^{j2\Pi (7/12)},\;0.5e^{j2\Pi (11/12)}\rangle }{u_{1}},\\ & \frac{\langle 0.1e^{j2\Pi (0)},\;0.3e^{j2\Pi (8/12)},\;0.9e^{j2\Pi (10/12)}\rangle }{u_{2}},\frac{\langle 0.9e^{j2\Pi (11/12)},\;0.2e^{j2\Pi (3/12)},\;0.06e^{j2\Pi (3/48)}\rangle }{u_{3}}\}),(\left(e_{4},p,0\right),\\ & \{\frac{\langle 0.9e^{j2\Pi (11/12)},\;0.5e^{j2\Pi (8/12)},\;0.1e^{j2\Pi (0)}\rangle }{u_{1}},\frac{\langle 0.8e^{j2\Pi (1)},\;0.5e^{j2\Pi (5/12)},\;0.06e^{j2\Pi (1/12)}\rangle }{u_{2}},\\ & \frac{\langle 0.09e^{j2\Pi (0)},\;0.9e^{j2\Pi (11/12)},\;1e^{j2\Pi (47/48)}\rangle }{u_{3}}\}),(\left(e_{4},q,0\right),\{\frac{\langle 0.5e^{j2\Pi (9/12)},\;0.7e^{j2\Pi (5/12)},\;0.2e^{j2\Pi (1/12)}\rangle }{u_{1}},\\ & \frac{\langle 0.9e^{j2\Pi (1)},\;0.7e^{j2\Pi (4/12)},\;0.1e^{j2\Pi (2/12)}\rangle }{u_{2}},\frac{\langle 0.06e^{j2\Pi (1/12)},\;0.8e^{j2\Pi (9/12)},\;0.9e^{j2\Pi (45/48)}\rangle }{u_{3}}\})\}. \end{array}$$

$$\begin{array}{cc} (G,B)= & \{(\left({\overset{´}{e}}_{1},p,1\right),\{\frac{\langle 0.2e^{j2\Pi (1/12)},\;0.1e^{j2\Pi (1/12)},\;0.7e^{j2\Pi (5/12)}\rangle }{u_{1}},\frac{\langle 0.9e^{j2\Pi (12/12)},\;0.3e^{j2\Pi (1/12)},\;0.1e^{j2\Pi (0)}\rangle }{u_{2}},\\ & \frac{\langle 0.5e^{j2\Pi (8/12)},\;0.1e^{j2\Pi (1/48)},\;0.5e^{j2\Pi (9/12)}\rangle }{u_{3}}\}),(\left({\overset{´}{e}}_{1},q,1\right),\{\frac{\langle 0.1e^{j2\Pi (1/12)},\;0.2e^{j\Pi (1/24)},\;0.6e^{j2\Pi (4/12)}\rangle }{u_{1}},\\ & \frac{\langle 0.7e^{j2\Pi (11/12)},\;0.3e^{j2\Pi (1/12)},\;0.2e^{j2\Pi (1/48)}\rangle }{u_{2}},\frac{\langle 0.4e^{j2\Pi (9/12)},\;0.1e^{j2\Pi (1/24)},\;0.7e^{j2\Pi (10/12)}\rangle }{u_{3}}\}),\\ & (\left({\overset{´}{e}}_{1},p,0\right),\{\frac{\langle 0.7e^{j2\Pi (11/12)},\;0.9e^{j2\Pi (11/12)},\;0.2e^{j2\Pi (7/12)}\rangle }{u_{1}},\frac{\langle 0.1e^{j2\Pi (0)},\;0.7e^{j2\Pi (11/12)},\;0.9e^{j2\Pi (12/12)}\rangle }{u_{2}},\\ & \frac{\langle 0.5e^{j2\Pi (4/12)},\;0.9e^{j2\Pi (47/48)},\;0.5e^{j2\Pi (3/12)}\rangle }{u_{3}}\}),(\left({\overset{´}{e}}_{1},q,0\right),\{\frac{\langle 0.6e^{j2\Pi (11/12)},\;0.8e^{j\Pi (23/24)},\;0.1e^{j2\Pi (8/12)}\rangle }{u_{1}},\\ & \frac{\langle 0.2e^{j2\Pi (1/12)},\;0.7e^{j2\Pi (11/12)},\;0.7e^{j2\Pi (47/48)}\rangle }{u_{2}},\frac{\langle 0.7e^{j2\Pi (3/12)},\;0.9e^{j2\Pi (23/24)},\;0.4e^{j2\Pi (2/12)}\rangle }{u_{3}}\}),(\left({\overset{´}{e}}_{2},p,1\right),\\ & \{\frac{\langle 0.2e^{j2\Pi (0)},\;0.1e^{j2\Pi (0)},\;0.5e^{j2\Pi (9/12)}\rangle }{u_{1}},\frac{\langle 1e^{j2\Pi (10/12)},\;0.01e^{j2\Pi (2/12)},\;0.03e^{j2\Pi (1/48)}\rangle }{u_{2}},\\ & \frac{\langle 0.1e^{j2\Pi (0)},\;0.1e^{j2\Pi (1/12)},\;0.2e^{j2\Pi (4/12)}\rangle }{u_{3}}\}),(\left({\overset{´}{e}}_{2},q,1\right),\{\frac{\langle 0.2e^{j2\Pi (1/48)},\;0.1e^{j2\Pi (0)},\;0.4e^{j2\Pi (9/12)}\rangle }{u_{1}},\\ & \frac{\langle 0.9e^{j2\Pi (11/12)},\;0.03e^{j2\Pi (2/12)},\;0.09e^{j2\Pi (1/24)}\rangle }{u_{2}},\frac{\langle 0.1e^{j2\Pi (0)},\;0.2e^{j2\Pi (3/12)},\;0.1e^{j2\Pi (3/12)}\rangle }{u_{3}}\}),(\left({\overset{´}{e}}_{2},p,0\right),\\ & \{\frac{\langle 0.5e^{j2\Pi (12/12)},\;0.9e^{j2\Pi (12/12)},\;0.2e^{j2\Pi (3/12)}\rangle }{u_{1}},\frac{\langle 0.03e^{j2\Pi (2/12)},\;0.99e^{j2\Pi (10/12)},\;1e^{j2\Pi (47/48)}\rangle }{u_{2}},\\ & \frac{\langle 0.2e^{j2\Pi (12/12)},\;0.9e^{j2\Pi (11/12)},\;0.1e^{j2\Pi (8/12)}\rangle }{u_{3}}\}),(\left({\overset{´}{e}}_{2},q,0\right),\{\frac{\langle 0.4e^{j2\Pi (47/48)},\;0.9e^{j2\Pi (12/12)},\;0.2e^{j2\Pi (3/12)}\rangle }{u_{1}},\\ & \frac{\langle 0.09e^{j2\Pi (1/12)},\;0.97e^{j2\Pi (10/12)},\;0.9e^{j2\Pi (23/24)}\rangle }{u_{2}},\frac{\langle 0.1e^{j2\Pi (12/12)},\;0.8e^{j2\Pi (9/12)},\;0.1e^{j2\Pi (9/12)}\rangle }{u_{3}}\}),(\left({\overset{´}{e}}_{3},p,1\right),\\ & \{\frac{\langle 1e^{j2\Pi (10/12)},\;0.02e^{j\Pi (0)},\;0.3e^{j2\Pi (1/12)}\rangle }{u_{1}},\frac{\langle 0.6e^{j2\Pi (9/12)},\;0.09e^{j2\Pi (1/12)},\;0.6e^{j2\Pi (7/12)}\rangle }{u_{2}},\\ & \frac{\langle 0.4e^{j2\Pi (0)},\;0.1e^{j2\Pi (1/12)},\;0.9e^{j2\Pi (7/12)}\rangle }{u_{3}}\}),(\left({\overset{´}{e}}_{3},q,1\right),\{\frac{\langle 0.8e^{j2\Pi (11/12)},\;0.1e^{j\Pi (0)},\;0.2e^{j2\Pi (4/12)}\rangle }{u_{1}},\\ & \frac{\langle 0.5e^{j2\Pi (7/12)},\;0.1e^{j2\Pi (3/12)},\;0.5e^{j2\Pi (8/12)}\rangle }{u_{2}},\frac{\langle 0.5e^{j2\Pi (0)},\;0.2e^{j2\Pi (3/12)},\;0.8e^{j2\Pi (8/12)}\rangle }{u_{3}}\}),(\left({\overset{´}{e}}_{3},p,0\right),\\ & \{\frac{\langle .03e^{j2\Pi (2/12)},\;0.98e^{j\Pi (1)},\;1e^{j2\Pi (11/12)}\rangle }{u_{1}},\frac{\langle 0.6e^{j2\Pi (3/12)},\;0.91e^{j2\Pi (11/12)},\;0.6e^{j2\Pi (5/12)}\rangle }{u_{2}},\\ & \frac{\langle 0.9e^{j2\Pi (1)},\;0.9e^{j2\Pi (11/12)},\;0.4e^{j2\Pi (5/12)}\rangle }{u_{3}}\}),(\left({\overset{´}{e}}_{3},q,0\right),\{\frac{\langle 0.2e^{j2\Pi (1/12)},\;0.9e^{j\Pi (1)},\;0.8e^{j2\Pi (8/12)}\rangle }{u_{1}},\\ & \frac{\langle 0.5e^{j2\Pi (5/12)},\;0.9e^{j2\Pi (9/12)},\;0.5e^{j2\Pi (4/12)}\rangle }{u_{2}},\frac{\langle 0.8e^{j2\Pi (1)},\;0.8e^{j2\Pi (9/12)},\;0.5e^{j2\Pi (4/12)}\rangle }{u_{3}}\}),(\left({\overset{´}{e}}_{4},p,1\right),\\ & \{\frac{\langle 0.2e^{j2\Pi (5/12)},\;0.7e^{j2\Pi (5/12)},\;0.1e^{j2\Pi (9/12)}\rangle }{u_{1}},\frac{\langle 0.2e^{j2\Pi (0)},\;0.3e^{j2\Pi (6/12)},\;0.9e^{j2\Pi (12/12)}\rangle }{u_{2}},\\ & \frac{\langle 1e^{j2\Pi (12/12)},\;0.02e^{j2\Pi (3/12)},\;0.1e^{j2\Pi (1/48)}\rangle }{u_{3}}\}),(\left({\overset{´}{e}}_{4},q,1\right),\{\frac{\langle 0.3e^{j2\Pi (6/12)},\;0.6e^{j2\Pi (3/12)},\;0.2e^{j2\Pi (7/12)}\rangle }{u_{1}},\\ & \frac{\langle 0.3e^{j2\Pi (0)},\;0.4e^{j2\Pi (7/12)},\;0.7e^{j2\Pi (11/12)}\rangle }{u_{2}},\frac{\langle 0.9e^{j2\Pi (11/12)},\;0.03e^{j2\Pi (4/12)},\;0.2e^{j2\Pi (2/48)}\rangle }{u_{3}}\}),(\left({\overset{´}{e}}_{4},p,0\right),\\ & \{\frac{\langle 0.1e^{j2\Pi (7/12)},\;0.3e^{j2\Pi (7/12)},\;0.2e^{j2\Pi (3/12)}\rangle }{u_{1}},\frac{\langle 0.9e^{j2\Pi (1)},\;0.7e^{j2\Pi (6/12)},\;0.2e^{j2\Pi (0)}\rangle }{u_{2}},\\ & \frac{\langle 0.1e^{j2\Pi (0)},\;0.98e^{j2\Pi (9/12)},\;1e^{j2\Pi (47/48)}\rangle }{u_{3}}\}),(\left({\overset{´}{e}}_{4},q,0\right),\{\frac{\langle 0.2e^{j2\Pi (6/12)},\;0.4e^{j2\Pi (9/12)},\;0.3e^{j2\Pi (5/12)}\rangle }{u_{1}},\\ & \frac{\langle 0.7e^{j2\Pi (1)},\;0.6e^{j2\Pi (5/12)},\;0.3e^{j2\Pi (1/12)}\rangle }{u_{2}},\frac{\langle 0.2e^{j2\Pi (1/12)},\;0.97e^{j2\Pi (7/12)},\;0.9e^{j2\Pi (46/48)}\rangle }{u_{3}}\})\}. \end{array}$$

*Now, we compute the relation between the two CNSESs (H,A) and (G,B) to investigate the effect of the volatility of exchange rates on the total exports of Malaysia. Our CNSER denoted by (R,C) where C⊆A×B such that (R,C)⊆(H,A)×(G,B) is:*

$$\begin{array}{ll} (R,C)= & \{(\left(e_{1},p,1\right),\left({\overset{´}{e}}_{1},p,1\right),\{\frac{\langle 0.01e^{j2\Pi (1/48)},\;0.2e^{j2\Pi (1/12)},\;0.9e^{j2\Pi (12/12)}\rangle }{u_{1}},\frac{\langle 0.9e^{j2\Pi (11/12)},\;0.3e^{j2\Pi (4/12)},\;0.1e^{j2\Pi (0)}\rangle }{u_{2}},\\ & \frac{\langle 0.04e^{j2\Pi (0)},\;0.2e^{j2\Pi (1/48)},\;0.7e^{j2\Pi (11/12)}\rangle }{u_{3}}\}),(\left(e_{1},q,1\right),\left({\overset{´}{e}}_{1},q,1\right),\{\frac{\langle 0.02e^{j2\Pi (0)},\;0.2e^{j\Pi (1/24)},\;0.8e^{j2\Pi (11/12)}\rangle }{u_{1}},\\ & \frac{\langle 0.7e^{j2\Pi (10/12)},\;0.3e^{j2\Pi (3/12)},\;0.2e^{j2\Pi (1/48)}\rangle }{u_{2}},\frac{\langle 0.05e^{j2\Pi (1/24)},\;0.4e^{j2\Pi (1/12)},\;0.7e^{j2\Pi (10/12)}\rangle }{u_{3}}\}),(\left(e_{1},p,0\right),\left({\overset{´}{e}}_{1},p,0\right),\\ & \{\frac{\langle 0.7e^{j2\Pi (11/12)},\;0.9e^{j2\Pi (23/24)},\;0.2e^{j2\Pi (7/12)}\rangle }{u_{1}},\frac{\langle 0.05e^{j2\Pi (0)},\;0.9e^{j2\Pi (11/12)},\;0.9e^{j2\Pi (12/12)}\rangle }{u_{2}},\\ & \frac{\langle 0.5e^{j2\Pi (4/12)},\;0.9e^{j2\Pi (47/48)},\;0.5e^{j2\Pi (3/12)}\rangle }{u_{3}}\}),(\left(e_{1},q,0\right),\left({\overset{´}{e}}_{1},q,0\right),\{\frac{\langle 0.6e^{j2\Pi (11/12)},\;0.9e^{j\Pi (23/24)},\;0.1e^{j2\Pi (8/12)}\rangle }{u_{1}},\\ & \frac{\langle 0.07e^{j2\Pi (1/12)},\;0.9e^{j2\Pi (11/12)},\;0.9e^{j2\Pi (47/48)}\rangle }{u_{2}},\frac{\langle 0.6e^{j2\Pi (3/12)},\;0.9e^{j2\Pi (23/24)},\;0.4e^{j2\Pi (2/12)}\rangle }{u_{3}}\}),(\left(e_{2},p,1\right),\left({\overset{´}{e}}_{2},p,1\right),\\ & \{\frac{\langle 0.05e^{j2\Pi (0)},\;0.1e^{j2\Pi (1/48)},\;0.7e^{j2\Pi (10/12)}\rangle }{u_{1}},\frac{\langle 0.9e^{j\Pi (9/12)},\;0.3e^{j2\Pi (2/12)},\;0.08e^{j2\Pi (1/48)}\rangle }{u_{2}},\\ & \frac{\langle 0.03e^{j2\Pi (0)},\;0.4e^{j2\Pi (1/12)},\;0.9e^{j2\Pi (11/12)}\rangle }{u_{3}}\}),(\left(e_{2},q,1\right),\left({\overset{´}{e}}_{2},q,1\right),\{\frac{\langle 0.02e^{j2\Pi (1/48)},\;0.2e^{j2\Pi (1/24)},\;0.8e^{j2\Pi (12/12)}\rangle }{u_{1}},\\ & \frac{\langle 0.8e^{j2\Pi (10/12)},\;0.3e^{j2\Pi (2/12)},\;0.08e^{j2\Pi (1/24)}\rangle }{u_{2}},\frac{\langle 0.06e^{j2\Pi (0)},\;0.2e^{j2\Pi (3/12)},\;0.9e^{j2\Pi (11/12)}\rangle }{u_{3}}\}),(\left(e_{2},p,0\right),\left({\overset{´}{e}}_{2},p,0\right),\\ & \{\frac{\langle 0.5e^{j2\Pi (12/12)},\;0.9e^{j2\Pi (12/12)},\;0.2e^{j2\Pi (3/12)}\rangle }{u_{1}},\frac{\langle 0.08e^{j\Pi (2/12)},\;0.99e^{j2\Pi (11/12)},\;1e^{j2\Pi (47/48)}\rangle }{u_{2}},\\ & \frac{\langle 0.2e^{j2\Pi (23/24)},\;0.9e^{j2\Pi (12/12)},\;0.1e^{j2\Pi (8/12)}\rangle }{u_{3}}\}),(\left(e_{2},q,0\right),\left({\overset{´}{e}}_{2},q,0\right),\{\frac{\langle 0.4e^{j2\Pi (23/24)},\;0.9e^{j2\Pi (12/12)},\;0.2e^{j2\Pi (3/12)}\rangle }{u_{1}},\\ & \frac{\langle 0.09e^{j2\Pi (1/12)},\;0.97e^{j2\Pi (10/12)},\;0.9e^{j2\Pi (47/48)}\rangle }{u_{2}},\frac{\langle 0.1e^{j2\Pi (12/12)},\;0.9e^{j2\Pi (47/48)},\;0.1e^{j2\Pi (9/12)}\rangle }{u_{3}}\}),(\left(e_{3},p,1\right),\left({\overset{´}{e}}_{3},p,1\right),\\ & \{\frac{\langle 0.9e^{j2\Pi (10/12)},\;0.1e^{j2\Pi (0)},\;0.3e^{j2\Pi (1/12)}\rangle }{u_{1}},\frac{\langle 0.01e^{j2\Pi (0)},\;0.1e^{j2\Pi (1/12)},\;0.7e^{j2\Pi (9/12)}\rangle }{u_{2}},\\ & \frac{\langle 0.05e^{j2\Pi (0)},\;0.4e^{j2\Pi (2/12)},\;0.9e^{j2\Pi (10/12)}\rangle }{u_{3}}\}),(\left(e_{3},q,1\right),\left({\overset{´}{e}}_{3},q,1\right),\{\frac{\langle 0.8e^{j2\Pi (10/12)},\;0.2e^{j2\Pi (1/48)},\;0.2e^{j2\Pi (4/12)}\rangle }{u_{1}},\\ & \frac{\langle 0.04e^{j2\Pi (0)},\;0.2e^{j2\Pi (3/12)},\;0.6e^{j2\Pi (10/12)}\rangle }{u_{2}},\frac{\langle 0.02e^{j2\Pi (0)},\;0.5e^{j2\Pi (3/12)},\;0.8e^{j2\Pi (11/12)}\rangle }{u_{3}}\}),(\left(e_{3},p,0\right),\left({\overset{´}{e}}_{3},p,0\right),\\ & \{\frac{\langle 0.02e^{j2\Pi (1/12)},\;0.98e^{j2\Pi (1)},\;1e^{j2\Pi (1)}\rangle }{u_{1}},\frac{\langle 0.6e^{j2\Pi (3/12)},\;0.91e^{j2\Pi (23/24)},\;0.6e^{j2\Pi (5/12)}\rangle }{u_{2}},\\ & \frac{\langle 0.6e^{j2\Pi (11/12)},\;0.9e^{j2\Pi (11/12)},\;0.4e^{j2\Pi (5/12)}\rangle }{u_{3}}\}),(\left(e_{3},q,0\right),\left({\overset{´}{e}}_{3},q,0\right),\{\frac{\langle 0.03e^{j2\Pi (1/12)},\;0.9e^{j2\Pi (1)},\;0.8e^{j2\Pi (1)}\rangle }{u_{1}},\\ & \frac{\langle 0.5e^{j2\Pi (5/12)},\;0.9e^{j2\Pi (21/24)},\;0.5e^{j2\Pi (4/12)}\rangle }{u_{2}},\frac{\langle 0.5e^{j2\Pi (9/12)},\;0.8e^{j2\Pi (11/12)},\;0.5e^{j2\Pi (4/12)}\rangle }{u_{3}}\}),(\left(e_{4},p,1\right),\left({\overset{´}{e}}_{4},p,1\right),\\ & \{\frac{\langle 0.1e^{j2\Pi (1/12)},\;0.7e^{j2\Pi (5/12)},\;0.9e^{j2\Pi (1)}\rangle }{u_{1}},\frac{\langle 0.06e^{j2\Pi (0)},\;0.5e^{j2\Pi (7/12)},\;0.9e^{j2\Pi (1)}\rangle }{u_{2}},\\ & \frac{\langle 1e^{j2\Pi (1)},\;0.1e^{j2\Pi (3/12)},\;0.1e^{j2\Pi (1/48)}\rangle }{u_{3}}\}),(\left(e_{4},q,1\right),\left({\overset{´}{e}}_{4},q,1\right),\{\frac{\langle 0.2e^{j2\Pi (3/12)},\;0.6e^{j2\Pi (7/12)},\;0.5e^{j2\Pi (11/12)}\rangle }{u_{1}},\\ & \frac{\langle 0.1e^{j2\Pi (0)},\;0.4e^{j2\Pi (8/12)},\;0.9e^{j2\Pi (11/12)}\rangle }{u_{2}},\frac{\langle 0.9e^{j2\Pi (11/12)},\;0.2e^{j2\Pi (4/12)},\;0.2e^{j2\Pi (3/48)}\rangle }{u_{3}}\}),(\left(e_{4},p,0\right),\left({\overset{´}{e}}_{4},p,0\right),\\ & \{\frac{\langle 0.1e^{j2\Pi (7/12)},\;0.5e^{j2\Pi (8/12)},\;0.2e^{j2\Pi (3/12)}\rangle }{u_{1}},\frac{\langle 0.8e^{j2\Pi (1)},\;0.7e^{j2\Pi (6/12)},\;0.2e^{j2\Pi (1/12)}\rangle }{u_{2}},\\ & \frac{\langle 0.09e^{j2\Pi (0)},\;0.98e^{j2\Pi (11/12)},\;1e^{j2\Pi (47/48)}\rangle }{u_{3}}\}),(\left(e_{4},q,0\right),\left({\overset{´}{e}}_{4},q,0\right),\{\frac{\langle 0.2e^{j2\Pi (6/12)},\;0.7e^{j2\Pi (9/12)},\;0.3e^{j2\Pi (5/12)}\rangle }{u_{1}},\\ & \frac{\langle 0.7e^{j2\Pi (1)},\;0.7e^{j2\Pi (5/12)},\;0.3e^{j2\Pi (2/12)}\rangle }{u_{2}},\frac{\langle 0.06e^{j2\Pi (1/12)},\;0.97e^{j2\Pi (9/12)},\;0.9e^{j2\Pi (46/48)}\rangle }{u_{3}}\})\}. \end{array}$$

In our example, the terms of the truth amplitude, indeterminate amplitude and false amplitude of (R,C) measure the membership degree of the impact of Malaysian exchange rate volatility on Malaysian exports, the indeterminacy membership degree of the impact of Malaysian exchange rate volatility on Malaysian exports and the non-membership degree of the impacts of Malaysian exchange rate volatility on Malaysian exports, respectively, whereas the truth phase term, the indeterminate phase term and the false phase term of (R,C) represent the period of time in which the Malaysian exchange rate variability influences the total of Malaysian exports, the period of time in which we are unable to determine if the Malaysian exchange rate variability influences or does not influence Malaysian exports and the period of time in which the Malaysian exchange rate variability does not influence Malaysian exports, respectively.

In the following discussion, we provide an example of scenarios that could possibly occur in this context. Let us consider the approximation:

$$\begin{array}{c} R(\left(e_{1},p,1\right),\left({\overset{´}{e}}_{1},p,1\right))=\;\\ \left(\{\frac{\langle 0.01e^{j2\Pi (1/48)},\;0.2e^{j2\Pi (1/12)},\;0.9e^{j2\Pi (12/12)}\rangle }{u_{1}},\frac{\langle 0.9e^{j2\Pi (11/12)},\;0.3e^{j2\Pi (4/12)},\;0.1e^{j2\Pi (0)}\rangle }{u_{2}},\frac{\langle 0.04e^{j2\Pi (0)},\;0.2e^{j2\Pi (1/48)},\;0.7e^{j2\Pi (11/12)}\rangle }{u_{3}}\}\right). \end{array}$$

For example, in the complex neutrosophic value $\frac{\langle 0.9e^{j2\Pi (11/12)},0.3e^{j2\Pi (4/12)},\;0.1e^{j2\Pi (0)}\rangle }{u_{2}}$, the complex-valued truth membership function $0.9e^{j2\Pi (11/12)}$ indicates that the expert *p* agrees that there is a strong influence of exchange rate volatility between MYR and USD on the total exports of Malaysia to the U.S., since the amplitude term 0.9 is very close to one and this influence span of 11 months is considered a very long time of influence (phase term with a value very close to one), and this influence is negative (an increase in variability between MYR and USD implies reduced Malaysia total export to the U.S.). The complex-valued indeterminacy membership function $0.3e^{j2\Pi (4/12)}$ can be interpreted as the expert *p* is unable to determine if there is a negative influence of exchange rate volatility on the total exports to the U.S. or not with a degree of 0.3, and this influence is not evident for four months. For the complex-valued false membership function $0.1e^{j2\Pi (0)}$, expert *p* is of the opinion that there is no negative influence with a degree of 0.1, and the time with no influence is zero.

Our problem is to measure the influence of the volatility of currency rates between the Malaysian ringgit (MYR) and U.S. dollar (USD), Chinese yuan (yuan), Japanese yen (JPY) and Singapore dollar (SD) on the total exports of Malaysia to these countries. Thus, this case study deals with four cases, and each case will be studied separately. For example, in the first case, we will determine if the total exports of Malaysia to the U.S. is affected negatively, positively or if it is not affected by the volatility of currency rates between the Malaysian ringgit (MYR) and U.S. dollar (USD). We will do the same with the remaining cases.

Next, the CNSER (R,C) is used together with a generalized algorithm to solve this MADM problem. In this algorithm, the first four steps will be applied to all cases. Step 5 to Step 8 deal with each case separately. The algorithm steps are given as follows.

**Step** **1:** Input the CNSESs (H,A) and (G,B).
**Step** **2:** Calculate The CNSER (R,C) of (H,A)×(G,B).
**Step** **3:** Convert the CNSER (R,C), which is actually a CNSES to the SVNSES $\left(\hat{R},C\right)$ by obtaining the weighted aggregation values of $T_{\hat{R}\left(\alpha ,\beta \right)}^{}\left(u\right),I_{\hat{R}\left(\alpha ,\beta \right)}^{}\left(u\right)$ and $F_{\hat{R}\left(\alpha ,\beta \right)}^{}\left(u\right)$, ∀(α,β)∈C and ∀u∈U as in the following formulas:
  $$T_{\hat{R}\left(\alpha ,\beta \right)}^{}\left(u\right)=w_{1}p_{R(\alpha ,\beta )}^{}\left(u\right)+w_{2}\left(1/2\pi \right)\mu_{R(\alpha ,\beta )}\left(u\right),$$
  $$I_{\hat{R}\left(\alpha ,\beta \right)}^{}\left(u\right)=w_{1}q_{R(\alpha ,\beta )}^{}\left(u\right)+w_{2}\left(1/2\pi \right)\nu_{R(\alpha ,\beta )}\left(u\right),$$
  $$F_{\hat{R}\left(\alpha ,\beta \right)}^{}\left(u\right)=w_{1}r_{R(\alpha ,\beta )}^{}\left(u\right)+w_{2}\left(1/2\pi \right)\omega_{R(\alpha ,\beta )}\left(u\right),$$
  where $p_{R(\alpha ,\beta )}^{}\left(u\right),q_{R(\alpha ,\beta )}^{}\left(u\right)$, $r_{R(\alpha ,\beta )}^{}\left(u\right)$ and $\mu_{R(\alpha ,\beta )}\left(u\right),\nu_{R(\alpha ,\beta )}\left(u\right),\omega_{R(\alpha ,\beta )}\left(u\right)$ are the amplitude and phase terms in the CNSES (R,C), respectively. $T_{\hat{R}\left(\alpha ,\beta \right)}^{}\left(u\right),I_{\hat{R}\left(\alpha ,\beta \right)}^{}\left(u\right)$ and $F_{\hat{R}\left(\alpha ,\beta \right)}^{}\left(u\right)$ are the truth membership function, indeterminacy membership function and falsity membership function in the SVNSES $\left(\hat{R},C\right)$, respectively, and $w_{1}$, $w_{2}$ are the weights for the amplitude terms (degrees of influence) and the phase terms (times of influence), respectively, where $w_{1}$ and $w_{2}\in \left[0,1\right]$ and $w_{1}+w_{2}=1.$
**Step** **4:** Find the values of $Z_{\hat{R}\left(\alpha ,\beta \right)}^{}\left(u\right)=\frac{T_{\hat{R}\left(\alpha ,\beta \right)}\left(u\right)+\left(1-I_{\hat{R}\left(\alpha ,\beta \right)}^{}\left(u\right)\right)+\left(1-F_{\hat{R}\left(\alpha ,\beta \right)}^{}\left(u\right)\right)}{3}$, ∀u∈U and ∀(α,β)∈C.
  Note that $Z_{\hat{R}\left(\alpha ,\beta \right)}^{}\left(u\right)$ comprises the normalized values of $S_{\hat{R}\left(\alpha ,\beta \right)}^{}\left(u\right)=T_{\hat{R}\left(\alpha ,\beta \right)}^{}\left(u\right)-I_{\hat{R}\left(\alpha ,\beta \right)}^{}\left(u\right)-F_{\hat{R}\left(\alpha ,\beta \right)}^{}\left(u\right),\forall u\in U$ and ∀(α,β)∈C. We normalize the elements of $S=\{S_{\hat{R}\left(\alpha ,\beta \right)}^{}\left(u\right),\forall u\in U$ and ∀(α,β)∈C}, since this represents the degree of the influence, where *S* takes its minimum value at −2 when $(T_{\hat{R}\left(\alpha ,\beta \right)}^{}\left(u\right),I_{\hat{R}\left(\alpha ,\beta \right)}^{}\left(u\right),F_{\hat{R}\left(\alpha ,\beta \right)}^{}\left(u\right))=\left(0,1,1\right)$, while its maximum takes the value one at $(T_{\hat{R}\left(\alpha ,\beta \right)}^{}\left(u\right),I_{\hat{R}\left(\alpha ,\beta \right)}^{}\left(u\right),F_{\hat{R}\left(\alpha ,\beta \right)}^{}\left(u\right))=\left(1,0,0\right)$.
**Step** **5:** Find the highest numerical grade of the elements in the agree-SVNSES and disagree-SVNSES for each ordered pair of the parameters $e_{j}$ and ${\overset{´}{e}}_{j}$.
**Step** **6:** Compute the score of each element $u_{i}\in U$ by taking the sum of the numerical grades of each element for the agree-SVNSES and disagree-SVNSES, denoted by $K_{i}$ and $S_{i}$, respectively.
**Step** **7:** Find the values of the score $r_{i}=K_{i}-S_{i}$ for each element $u_{i}\;\in U$.
**Step** **8:** Determine the value of the highest score $m=max_{u_{i\;\in U}}\left\{r_{i}\right\}$. The decision is to choose the associated element $u_{i}$ as the appropriate solution under the the parameter $e_{j}$ and its corresponding ${\overset{´}{e}}_{j}$.

It is to be noted that this method is used to deal with decision-making problems with known weight information (complete weight information). To execute the above steps, we assume that the weight vector for the amplitude terms is $w_{1}=0.6$ and the weight vector for the phase terms is $w_{2}=0.4$

Now, to convert the CNSES (R,C) to the SVNSES $\left(\hat{R},C\right)$, obtain the weighted aggregation values of $T_{\hat{R}\left(\alpha ,\beta \right)}^{}\left(u\right),I_{\hat{R}\left(\alpha ,\beta \right)}^{}\left(u\right)$ and $F_{\hat{R}\left(\alpha ,\beta \right)}^{}\left(u\right)$,∀(α,β)∈C and ∀u∈U. To illustrate this step, we calculate $T_{\hat{R}\left(\alpha ,\beta \right)}^{}\left(u_{}\right),I_{\hat{R}\left(\alpha ,\beta \right)}^{}\left(u_{}\right)$ and $F_{\hat{R}\left(\alpha ,\beta \right)}^{}\left(u_{}\right)$, when $\left(\alpha ,\beta \right)=(\left(e_{1},p,1\right),\left({\overset{´}{e}}_{1},p,1\right))$ and $u=u_{1}$ as shown below:

$$\begin{array}{cc} T_{\hat{R}\left(\left(e_{1},p,1\right),\left({\overset{´}{e}}_{1},p,1\right)\right)}^{}\left(u_{1}\right) & =w_{1}p_{R(\left(e_{1},p,1\right),\left({\overset{´}{e}}_{1},p,1\right))}^{}\left(u_{1}\right)+w_{2}\left(1/2\pi \right)\mu_{R(\left(e_{1},p,1\right),\left({\overset{´}{e}}_{1},p,1\right))}\left(u_{1}\right)\\ & =0.6(0.01)+0.4(1/2\pi )(2\pi )(1/48)\\ & =0.014. \end{array}$$

$$\begin{array}{cc} I_{\hat{R}\left(\left(e_{1},p,1\right),\left({\overset{´}{e}}_{1},p,1\right)\right)}^{}\left(u_{1}\right) & =w_{1}q_{R(\left(e_{1},p,1\right),\left({\overset{´}{e}}_{1},p,1\right))}^{}\left(u_{1}\right)+w_{2}\left(1/2\pi \right)\nu_{R(\left(e_{1},p,1\right),\left({\overset{´}{e}}_{1},p,1\right))}\left(u_{1}\right)\\ & =0.6(0.2)+0.4(1/2\pi )(2\pi )(1/12)\\ & =0.153. \end{array}$$

$$\begin{array}{cc} F_{\hat{R}\left(\left(e_{1},p,1\right),\left({\overset{´}{e}}_{1},p,1\right)\right)}^{}\left(u_{1}\right) & =w_{1}r_{R(\left(e_{1},p,1\right),\left({\overset{´}{e}}_{1},p,1\right))}^{}\left(u_{1}\right)+w_{2}\left(1/2\pi \right)\omega_{R(\left(e_{1},p,1\right),\left({\overset{´}{e}}_{1},p,1\right))}\left(u_{1}\right)\\ & =0.6(0.9)+0.4(1/2\pi )(2\pi )(1)\\ & =0.94. \end{array}$$

Then, for $\left(\alpha ,\beta \right)=(\left(e_{1},p,1\right),\left({\overset{´}{e}}_{1},p,1\right))$ and $u=u_{1}$, the single-valued neutrosophic soft expert value (SVNSEV) is $\left(T_{\hat{R}\left(\alpha ,\beta \right)}^{}\left(u_{}\right),I_{\hat{R}\left(\alpha ,\beta \right)}^{}\left(u_{}\right),F_{\hat{R}\left(\alpha ,\beta \right)}^{}\left(u_{}\right))=(0.014,0.153,0.94\right)$.

In the same manner, we calculate the other SVNSEVs, ∀(α,β)∈C and ∀u∈U as in Table 1, which gives the values of $Z_{\hat{R}\left(\alpha ,\beta \right)}^{}\left(u\right),\forall \left(\alpha ,\beta \right)\in C$ and ∀u∈U.

It is to be noted that the upper and lower terms for each element in Table 1 represent the SVNSEVs, ∀(α,β)∈C and ∀u∈U and the values of $Z_{\hat{R}\left(\alpha ,\beta \right)}^{}\left(u\right),\forall \left(\alpha ,\beta \right)\in C$ and ∀u∈U, respectively. Now, we apply Step 5 to Step 8 to each case separately. We begin by examining the first case to determine the type of relationship between the total exports of Malaysia to the U.S. and the variability of currency rates between MYR and USD.

Table 2 and Table 3 give the highest numerical grade for the elements in the agree-SVNSES and disagree-SVNSES, respectively, under the the parameter $e_{1}$ and its corresponding ${\overset{´}{e}}_{1}$.

Let $K_{i}$ and $S_{i}$ represent the score of each numerical grade for the agree-SVNSES and disagree-SVNSES, respectively. These values are given in Table 4.

From Table 4, $max_{u_{i}\in U}\left\{r_{i}\right\}$ = $r_{2}$. Thus, the decision is to choose the associated element $u_{2}$, which represents “negative influence”, as the appropriate solution under the the parameter $e_{1}$ and its corresponding ${\overset{´}{e}}_{1}$. Therefore, we conclude that the total of the exports of Malaysia to the U.S. is affected negatively by the variability of currency rates between MYR and USD.

With regard to Malaysia’s export to China, Japan and Singapore, we use the same calculations above. In the case of China, we obtain $u_{2}$ as the optimal solution under the the parameter $e_{2}$ and its corresponding ${\overset{´}{e}}_{2}$, which means that the total of the exports of Malaysia to China is affected negatively by the variability of currency rates between MYR and yuan. In contrast with the Malaysia export to the U.S. and China, we find that the variability of currency rates between MYR and JPY affects the total of the exports of Malaysia to Japan positively ($u_{1}$ gained the highest score under parameters $e_{3}$ and ${\overset{´}{e}}_{3}$). Finally, we conclude that there is no interaction between the total exports of Malaysia to Singapore and the variability of exchange rates between MYR and SD as $u_{3}$ gets the highest score under parameters $e_{4}$ and ${\overset{´}{e}}_{4}$. Most probably, there has been a commercial exchange between Malaysia and Singapore due to many factors such as geography, history, politics, ideology, culture and ethnicity that surpasses the factor of the currency rate alone.

In general, we conclude that the influence of currency rates’ variability on Malaysian exports is open to interpretation and differs from country to country based on the nature of the relation between Malaysia and those countries.

### 3.2. Comparison between CNSER and the Existing Models

We have used the CNSER to determine the influence of the volatility of currency rates on the total exports of Malaysia, where its amplitude terms represent the degree of the influence and its phase terms represent the total time of the influence. In this section, we will compare our proposed CNSER model to two other existing models, neutrosophic soft relations [34] and complex Atanassov’s intuitionistic fuzzy relation (CAIFR) [35].

As an extension of fuzzy soft relation [32] and intuitionistic fuzzy soft relation [33], neutrosophic soft relation [34] was developed to measure the degree of the interaction between two neutrosophic soft sets, with the ability to handle all types of uncertainties including indeterminacy which is beyond the scope of fuzzy and intuitionistic fuzzy soft relations. However, neutrosophic soft relation fails to deal with problems that involve two-dimensional information/date i.e., two different types of information/data pertaining to the problem parameters.

From Example 2, it can be seen that neutrosophic soft relation is not able to solve the decision making problem presented, which involves two types of information (the degree of the influence and the total time of the influence) since neutrosophic soft relation lacks the phase terms which represent the time frame of this problem. An additional reason, is its inability to deal with more than one expert.

CAIFR [35] can overcome the problems inherent in using neutrosophic soft sets by virtue of the phase terms which have the ability to represent the time frame of the interaction between the variables. However, the complex Atanassov’s intuitionistic fuzzy model has some deficiencies compared to CNSER. Firstly, it does not have the parameterizations tool to describe the relation variables in a complete and comprehensive way, unlike CNSER which has the added advantage of soft sets. Secondly, CAIFR [35] is characterized by complex-valued truth membership and complex-valued falsity membership functions which handle only incomplete and uncertainty information, whereas CNSER can handle all types of uncertainty including indeterminacy with complex-valued truth membership function, complex-valued indeterminacy membership function and complex-valued falsity membership function. Finally, CAIFR does not have a mechanism to incorporate the opinion of all experts in one model which is a major problem in any relation that is used to handle and model uncertainties and indeterminacies, as most real-life situations are often ambiguous. CNSER addresses this problem, by providing the opinions of the experts. This makes it more valid and real in modeling real life problems where the judgments of human beings play a major role.

## 4. Operations on Complex Neutrosophic Soft Expert Relation

We will now introduce some basic operations on CNSER such as the inverse, complement and composition of CNSERs. We will begin by first proposing the definition of the complement of CNSER.

**Definition** **8.** *Let (R,C) be a CNSER from (H,A) to (G,B), where $\left(R,C\right)=\left\{\langle \left(\alpha ,\beta \right),T_{R(\alpha ,\beta )}\left(u\right),I_{R(\alpha ,\beta )}\left(u\right),F_{R(\alpha ,\beta )}\left(u\right)\rangle :\left(\alpha ,\beta \right)\in C\subseteq A\times B,u\in U\right\}.$ The complement of (R,C) is defined as follows.*

$${\left(R,C\right)}^{c}=\left\{\langle \left(\alpha ,\beta \right),T_{R^{c}\left(\alpha ,\beta \right)}\left(u\right),I_{R^{c}\left(\alpha ,\beta \right)}\left(u\right),F_{R^{c}\left(\alpha ,\beta \right)}\left(u\right)\rangle :\left(\alpha ,\beta \right)\in C\subseteq A\times B,u\in U\right\},$$

*where ∀(α,β)∈C⊆A×B and ∀u∈U,*

$$T_{R^{c}\left(\alpha ,\beta \right)}\left(u\right)=c\left(p_{R(\alpha ,\beta )}\left(u\right)\right).e^{j\mu_{R(\alpha ,\beta )}^{c}\left(u\right)}=r_{R(\alpha ,\beta )}\left(u\right).e^{j(2\pi -\mu_{R(\alpha ,\beta )}\left(u\right))},$$

$$I_{R^{c}\left(\alpha ,\beta \right)}\left(u\right)=c\left(q_{R(\alpha ,\beta )}\left(u\right)\right).e^{j\nu_{R(\alpha ,\beta )}^{c}\left(u\right)}=\left(1-q_{R(\alpha ,\beta )}\left(u\right)\right).e^{j(2\pi -\nu_{R(\alpha ,\beta )}\left(u\right))},$$

$$F_{R^{c}\left(\alpha ,\beta \right)}\left(u\right)=c\left(r_{R(\alpha ,\beta )}\left(u\right)\right).e^{j\omega_{R(\alpha ,\beta )}^{c}\left(u\right)}=p_{R(\alpha ,\beta )}\left(u\right).e^{j(2\pi -\omega_{R(\alpha ,\beta )}\left(u\right))}.$$

Next, we will give the definition of the inverse of a CNSER and give a proposition of the inverse of a CNSER.

**Definition** **9.** *Let (R,C) be a CNSER from (H,A) to (G,B) where C⊆A×B. The inverse of (R,C) is denoted as ${\left(R,C\right)}^{-1}$ and is a CNSER from (G,B) to (H,A) defined as:*

$$R^{-1}\left(\alpha ,\beta \right)=R\left(\beta ,\alpha \right),\forall \left(\alpha ,\beta \right)\in C\subseteq A\times B.$$

**Proposition** **1.** *Let U be a universe, (H,A) and (G,B) be two CNSESs over U and suppose that $\left(R_{1},C\right)$ and $\left(R_{2},C\right)$ are two CNSERs from (H,A) to (G,B), where C⊆A×B. Then, the following results hold true:*

*1.*  ${\left({\left(R_{1},C\right)}^{-1}\right)}^{-1}=\left(R_{1},C\right).$
*2.*  If $\left(R_{1},C\right)\subseteq \left(R_{2},C\right)$ then ${\left(R_{1},C\right)}^{-1}\subseteq {\left(R_{2},C\right)}^{-1}.$

**Proof.**  ∀(α,β)∈C⊆A×B, we have

1. ${\left(R_{1}^{-1}\right)}^{-1}\left(\alpha ,\beta \right)=R_{1}^{-1}\left(\beta ,\alpha \right)=R_{1}\left(\alpha ,\beta \right)$, thus ${\left({\left(R_{1},C\right)}^{-1}\right)}^{-1}=\left(R_{1},C\right)$.
2. If $R_{1}\left(\alpha ,\beta \right)\subseteq R_{2}\left(\alpha ,\beta \right)$, then $R_{1}^{-1}\left(\beta ,\alpha \right)\subseteq R_{2}^{-1}\left(\beta ,\alpha \right)$ and, thus, ${\left(R_{1},C\right)}^{-1}\subseteq {\left(R_{2},C\right)}^{-1}.$

 ☐

Next, we will propose the axiomatic definition of the composition of CNSERs along with an illustrative example, followed by two associated theorems.

**Definition** **10.** Let (H,A),(G,B) and (K,C) be three complex neutrosophic soft expert sets over a universe U, $\left(R_{1},D_{1}\right)$ be a CNSER from (H,A) to (G,B) and $\left(R_{2},D_{2}\right)$ be a CNSER from (G,B) to (K,C), where $D_{1}\subseteq A\times B$ and $D_{2}\subseteq B\times C.$ The composition of the complex neutrosophic soft expert relations $\left(R_{1},D_{1}\right)$ and $\left(R_{2},D_{2}\right)$ is defined as: $\left(R_{1}\circ R_{2}\right)\left(a,c\right)=\left\{\langle \left(a,c\right),T_{R_{1}\circ R_{2}\left(a,c\right)}\left(u\right),I_{R_{1}\circ R_{2}\left(a,c\right)}\left(u\right),F_{R_{1}\circ R_{2}\left(a,c\right)}\left(u\right)\rangle :\left(a,c\right)\in A\times C,u\in U\right\},$ where $\forall \left(a,b\right)\in D_{1}\subseteq A\times B$ and $\left(b,c\right)\in D_{2}\subseteq B\times C,$$T_{R_{1}\circ R_{2}\left(a,c\right)}\left(u\right)=p_{R_{1}\circ R_{2}\left(a,c\right)}\left(u\right).e^{j\mu_{R_{1}\circ R_{2}\left(a,c\right)}\left(u\right)}$, where $p_{R_{1}\circ R_{2}\left(a,c\right)}\left(u\right)=max\left[p_{R_{1}\left(a,b\right)}\left(u\right),p_{R_{2}\left(b,c\right)}\left(u\right)\right]=max[min\left(p_{H(a)}\left(u\right),p_{G(b)}\left(u\right)\right),min(p_{G(b)}\left(u\right)$, $p_{K(c)}\left(u\right))]$ and $\mu_{R_{1}\circ R_{2}\left(a,c\right)}\left(u\right)=max\left[\mu_{R_{1}\left(a,b\right)}\left(u\right),\mu_{R_{2}\left(b,c\right)}\left(u\right)\right]=max[min\left(\mu_{H(a)}\left(u\right),\mu_{G(b)}\left(u\right)\right),min\left(\mu_{G(b)}\left(u\right),\mu_{K(c)}\left(u\right)\right)]$, $I_{R_{1}\circ R_{2}\left(a,c\right)}\left(u\right)=q_{R_{1}\circ R_{2}\left(a,c\right)}\left(u\right).e^{j\nu_{R_{1}\circ R_{2}\left(a,c\right)}\left(u\right)}$, where $q_{R_{1}\circ R_{2}\left(a,c\right)}\left(u\right)=min\left[q_{R_{1}\left(a,b\right)}\left(u\right),q_{R_{2}\left(b,c\right)}\left(u\right)\right]=min[max\left(q_{H(a)}\left(u\right),q_{G(b)}\left(u\right)\right),max(q_{G(b)}\left(u\right)$, $q_{K(c)}\left(u\right))]$ and $\nu_{R_{1}\circ R_{2}\left(a,c\right)}\left(u\right)=min\left[\nu_{R_{1}\left(a,b\right)}\left(u\right),\nu_{R_{2}\left(b,c\right)}\left(u\right)\right]=min[max\left(\nu_{H(a)}\left(u\right),\nu_{G(b)}\left(u\right)\right),max\left(\nu_{G(b)}\left(u\right),\nu_{K(c)}\left(u\right)\right)]$, $F_{R_{1}\circ R_{2}\left(a,c\right)}\left(u\right)=r_{R_{1}\circ R_{2}\left(a,c\right)}\left(u\right).e^{j\omega_{R_{1}\circ R_{2}\left(a,c\right)}\left(u\right)}$, where $r_{R_{1}\circ R_{2}\left(a,c\right)}\left(u\right)=max\left[r_{R_{1}\left(a,b\right)}\left(u\right),r_{R_{2}\left(b,c\right)}\left(u\right)\right]=max[min\left(r_{H(a)}\left(u\right),r_{G(b)}\left(u\right)\right),min\left(r_{G(b)}\left(u\right),r_{K(c)}\left(u\right)\right)]$ and $\omega_{R_{1}\circ R_{2}\left(a,c\right)}\left(u\right)=max\left[\omega_{R_{1}\left(a,b\right)}\left(u\right),\omega_{R_{2}\left(b,c\right)}\left(u\right)\right]=max[min\left(\omega_{H(a)}\left(u\right),\omega_{G(b)}\left(u\right)\right),min\left(\omega_{G(b)}\left(u\right),\omega_{K(c)}\left(u\right)\right)]$.

This relationship can be written as $\left(R_{1}\circ R_{2}\right)\left(a,c\right)=R_{1}\left(a,b\right){\tilde{\cap }}_{N}R_{2}\left(b,c\right)$.

In the following, we give an example of the composition of complex neutrosophic soft expert relations to illustrate how to employ both components of the amplitude term and phase term to convey the idea of the composition concept.

**Example** **3.** *Suppose that $U=\{u_{1},u_{2},u_{3}\}$ is a set of medication and $E=\{a_{1},a_{2},b_{1},b_{2},c_{1},c_{2}\}$ is a set of parameters that represent a set of diseases, where $a_{1}$ = dengue, $a_{2}$ = malaria, $b_{1}=$ chikungunya, $b_{2}$ = cholera, $c_{1}=$ typhoid and $c_{2}$ = yellow fever. Let X={p,q} be a set of experts who are assigned to give their opinions concerning these medications by taking into account both the degree of effectiveness of a medicine on a disease and the time that is taken by this medicine to overcome this disease. Let (H,A), (G,B) and (K,C) be three CNSESs over U, where $A=\{\left(a_{1},p,1\right),\left(a_{2},q,1\right)\}$, $B=\{\left(b_{1},p,1\right),\left(b_{2},p,0\right)\}$ and $C=\{\left(c_{1},q,0\right),\left(c_{2},p,1\right)\}$. Then, the CNSESs (H,A), (G,B) and (K,C) are defined as follows:*

$$\begin{array}{cc} (H,A)= & \{\left(\left(a_{1},p,1\right),\left\{\frac{\langle 0.4e^{j0.9\Pi },\;0.6e^{j0.1\Pi },\;0.6e^{j0.1\Pi }\rangle }{u_{1}},\frac{\langle 0.8e^{j0.5\Pi },\;0.7e^{j0.3\Pi },\;0.8e^{j0.7\Pi }\rangle }{u_{2}},\frac{\langle 0.4e^{j0.9\Pi },\;0.4e^{j0.4\Pi },\;0.1e^{j0.7\Pi }\rangle }{u_{3}}\right\}\right),\\ & \left(\left(a_{2},q,1\right),\left\{\frac{\langle 0.5e^{j0.3\Pi },\;0.5e^{j0.2\Pi },\;0.2e^{j0.2\Pi }\rangle }{u_{1}},\frac{\langle 0.8e^{j0.8\Pi },\;0.1e^{j0.4\Pi },\;0.7e^{j0.3\Pi }\rangle }{u_{2}},\frac{\langle 0.6e^{j0.9\Pi },\;0.8e^{j0.9\Pi },\;0.4e^{j0.7\Pi }\rangle }{u_{3}}\right\}\right)\}. \end{array}$$

$$\begin{array}{cc} (G,B)= & \{\left(\left(b_{1},p,1\right),\left\{\frac{\langle 0.1e^{j0.5\Pi },\;0.4e^{j0.9\Pi },\;0.2e^{j0.2\Pi }\rangle }{u_{1}},\frac{\langle 0.1e^{j0.6\Pi },\;0.7e^{j0.4\Pi },\;0.2e^{j0.7\Pi }\rangle }{u_{2}},\frac{\langle 0.5e^{j0.8\Pi },\;0.3e^{j0.5\Pi },\;0.6e^{j0.9\Pi }\rangle }{u_{3}}\right\}\right),\\ & \left(\left(b_{2},p,0\right),\left\{\frac{\langle 0.9e^{j0.4\Pi },\;0.4e^{j0.8\Pi },\;0.7e^{j0.3\Pi }\rangle }{u_{1}},\frac{\langle 0.6e^{j0.4\Pi },\;0.5e^{j0.7\Pi },\;0.8e^{j0.6\Pi }\rangle }{u_{2}},\frac{\langle 0.3e^{j0.8\Pi },\;0.4e^{j0.8\Pi },\;0.2e^{j0.1\Pi }\rangle }{u_{3}}\right\}\right)\}. \end{array}$$

$$\begin{array}{cc} (K,C)= & \{\left(\left(c_{1},q,0\right),\left\{\frac{\langle 0.7e^{j0.1\Pi },\;0.1e^{j0.5\Pi },\;0.3e^{j0.4\Pi }\rangle }{u_{1}},\frac{\langle 0.7e^{j0.4\Pi },\;0.2e^{j0.3\Pi },\;0.3e^{j0.4\Pi }\rangle }{u_{2}},\frac{\langle 0.1e^{j0.5\Pi },\;0.8e^{j0.6\Pi },\;0.9e^{j0.1\Pi }\rangle }{u_{3}}\right\}\right),\\ & \left(\left(c_{2},p,1\right),\left\{\frac{\langle 0.4e^{j0.2\Pi },\;0.6e^{j0.2\Pi },\;0.1e^{j0.2\Pi }\rangle }{u_{1}},\frac{\langle 0.2e^{j0.7\Pi },\;0.3e^{j0.4\Pi },\;0.6e^{j0.5\Pi }\rangle }{u_{2}},\frac{\langle 0.9e^{j0.2\Pi },\;0.1e^{j0.7\Pi },\;0.9e^{j0.6\Pi }\rangle }{u_{3}}\right\}\right)\}. \end{array}$$

*The CNSERs $\left(R_{1},D_{1}\right)$ from (H,A) to (G,B) and $\left(R_{2},D_{2}\right)$ from (G,B) to (K,C) are defined below if and only if a, b and c are illnesses carried by mosquitoes, $\forall \left(a,b\right)\in D_{1}\subseteq A\times B$ and $\left(b,c\right)\in D_{2}\subseteq B\times C,$ respectively. Then, the CNSERs $\left(R_{1},D_{1}\right)$ and $\left(R_{2},D_{2}\right)$ are given by:*

$$\begin{array}{cc} (R_{1},D_{1})= & \{\left(\left(\left(a_{1},p,1\right),\left(b_{1},p,1\right)\right),\left\{\frac{\langle 0.1e^{j0.5\Pi },\;0.6e^{j0.9\Pi },\;0.6e^{j0.2\Pi }\rangle }{u_{1}},\frac{\langle 0.1e^{j0.5\Pi },\;0.7e^{j0.4\Pi },\;0.8e^{j0.7\Pi }\rangle }{u_{2}},\frac{\langle 0.4e^{j0.8\Pi },\;0.4e^{j0.5\Pi },\;0.6e^{j0.9\Pi }\rangle }{u_{3}}\right\}\right),\\ & \left(\left(\left(a_{2},q,1\right),\left(b_{1},p,1\right)\right),\left\{\frac{\langle 0.1e^{j0.3\Pi },\;0.5e^{j0.9\Pi },\;0.2e^{j0.2\Pi }\rangle }{u_{1}},\frac{\langle 0.1e^{j0.6\Pi },\;0.7e^{j0.4\Pi },\;0.7e^{j0.7\Pi }\rangle }{u_{2}},\frac{\langle 0.5e^{j0.8\Pi },\;0.8e^{j0.9\Pi },\;0.6e^{j0.9\Pi }\rangle }{u_{3}}\right\}\right)\}. \end{array}$$

$$\begin{array}{cc} (R_{2},D_{2})= & \left\{\left(\left(\left(b_{1},p,1\right),\left(c_{2},p,1\right)\right),\left\{\frac{\langle 0.1e^{j0.2\Pi },\;0.6e^{j0.9\Pi },\;0.2e^{j0.2\Pi }\rangle }{u_{1}},\frac{\langle 0.1e^{j0.6\Pi },\;0.7e^{j0.4\Pi },\;0.6e^{j0.7\Pi }\rangle }{u_{2}},\frac{\langle 0.5e^{j0.2\Pi },\;0.3e^{j0.7\Pi },\;0.9e^{j0.9\Pi }\rangle }{u_{3}}\right\}\right)\right\}. \end{array}$$

*The composition between the CNSERs $\left(R_{1},D_{1}\right)$ and $\left(R_{2},D_{2}\right)$ is as follows.*

$$\begin{array}{cc} \left(R_{1}\circ R_{2}\right)\left(a,c\right)= & \{\left(\left(\left(a_{1},p,1\right),\left(c_{2},p,1\right)\right),\left\{\frac{\langle 0.1e^{j0.5\Pi },\;0.6e^{j0.9\Pi },\;0.2e^{j0.2\Pi }\rangle }{u_{1}},\frac{\langle 0.1e^{j0.6\Pi },\;0.7e^{j0.4\Pi },\;0.6e^{j0.7\Pi }\rangle }{u_{2}},\frac{\langle 0.5e^{j0.8\Pi },\;0.3e^{j0.5\Pi },\;0.6e^{j0.9\Pi }\rangle }{u_{3}}\right\}\right),\;\\ & \left(\left(\left(a_{2},q,1\right),\left(c_{2},p,1\right)\right),\left\{\frac{\langle 0.1e^{j0.3\Pi },\;0.5e^{j0.9\Pi },\;0.2e^{j0.2\Pi }\rangle }{u_{1}},\frac{\langle 0.1e^{j0.6\Pi },\;0.7e^{j0.4\Pi },\;0.6e^{j0.7\Pi }\rangle }{u_{2}},\frac{\langle 0.5e^{j0.8\Pi },\;0.3e^{j0.7\Pi },\;0.6e^{j0.9\Pi }\rangle }{u_{3}}\right\}\right)\}. \end{array}$$

**Theorem** **1.** Let $\left(R_{1},D_{1}\right)$ be a CNSER between (H,A) and (G,B) and $\left(R_{2},D_{2}\right)$ be a CNSER between (G,B) and (K,C), such that $D_{1}\subseteq A\times B$, $D_{2}\subseteq B\times C$ and (H,A), (G,B) and (K,C) are CNSESs over a soft universe (U,E), then ${\left(R_{1}\circ R_{2}\right)}^{-1}=R_{2}^{-1}\circ R_{1}^{-1}.$

**Proof.**  $\forall \left(a,b\right)\in D_{1}\subseteq A\times B$ and $\left(b,c\right)\in D_{2}\subseteq B\times C,$ let ${\left(R_{1}\circ R_{2}\right)}^{-1}\left(a,c\right)=\left\{\langle \left(a,c\right),T_{{\left(R_{1}\circ R_{2}\right)}^{-1}\left(a,c\right)}\left(u\right),I_{{\left(R_{1}\circ R_{2}\right)}^{-1}\left(a,c\right)}\left(u\right),F_{{\left(R_{1}\circ R_{2}\right)}^{-1}\left(a,c\right)}\left(u\right)\rangle :\left(a,c\right)\in D_{1}\times D_{2}\right\}$ and $R_{2}^{-1}\circ R_{1}^{-1}\left(a,c\right)=\left\{\langle \left(a,c\right),T_{R_{2}^{-1}\circ R_{1}^{-1}\left(a,c\right)}\left(u\right),I_{R_{2}^{-1}\circ R_{1}^{-1}\left(a,c\right)}\left(u\right),F_{R_{2}^{-1}\circ R_{1}^{-1}\left(a,c\right)}\left(u\right)\rangle :\left(a,c\right)\in D_{1}\times D_{2}\right\}.$

To prove the equality, we have to show that $T_{{\left(R_{1}\circ R_{2}\right)}^{-1}\left(a,c\right)}\left(u\right)=T_{R_{2}^{-1}\circ R_{1}^{-1}\left(a,c\right)}\left(u\right)$, $I_{{\left(R_{1}\circ R_{2}\right)}^{-1}\left(a,c\right)}\left(u\right)=I_{R_{2}^{-1}\circ R_{1}^{-1}\left(a,c\right)}\left(u\right)$ and $F_{{\left(R_{1}\circ R_{2}\right)}^{-1}\left(a,c\right)}\left(u\right)=F_{R_{2}^{-1}\circ R_{1}^{-1}\left(a,c\right)}\left(u\right)$. Therefore,

$$\begin{array}{cc} p_{{\left(R_{1}\circ R_{2}\right)}^{-1}\left(a,c\right)}\left(u\right) & =p_{\left(R_{1}\circ R_{2}\right)\left(c,a\right)}\left(u\right)\\ & =max\left(p_{R_{1}\left(c,b\right)}\left(u\right),p_{R_{2}\left(b,a\right)}\left(u\right)\right),=max\left(p_{R_{2}\left(b,a\right)}\left(u\right),p_{R_{1}\left(c,b\right)}\left(u\right)\right),\\ & =max[min\left(p_{G(b)}\left(u\right),p_{H(a)}\left(u\right)\right),min\left(p_{k(c)}\left(u\right),p_{G(b)}\left(u\right)\right)],\\ & =max[min\left(p_{H(a)}\left(u\right),p_{G(b)}\left(u\right)\right),min\left(p_{G(b)}\left(u\right)\right),p_{k(c)}\left(u\right)],\\ & =max(p_{R_{2}^{-1}\left(a,b\right)}\left(u\right),p_{R_{1}^{-1}\left(b,c\right)}\left(u\right)),\\ & =p_{R_{2}^{-1}\circ R_{1}^{-1}\left(a,c\right)}\left(u\right), \end{array}$$

which implies $p_{{\left(R_{1}\circ R_{2}\right)}^{-1}\left(a,c\right)}\left(u\right)=p_{R_{2}^{-1}\circ R_{1}^{-1}\left(a,c\right)}\left(u\right)$. Similarly, we can show that $\mu_{{\left(R_{1}\circ R_{2}\right)}^{-1}\left(a,c\right)}\left(u\right)=\mu_{R_{2}^{-1}\circ R_{1}^{-1}\left(a,c\right)}\left(u\right)$, proving that $T_{{\left(R_{1}\circ R_{2}\right)}^{-1}\left(a,c\right)}\left(u\right)=T_{R_{2}^{-1}\circ R_{1}^{-1}\left(a,c\right)}\left(u\right)$. The proofs for the identity and falsity terms can be similarly proven. ☐

**Theorem** **2.** Let $\left(R_{1},E_{1}\right)$ be a CNSER between (H,A) and $\left(G,B),\;(R_{2},E_{2}\right)$ a CNSER between (G,B) and (K,C) and $\left(R_{3},E_{3}\right)$ a CNSER between (K,C) and (L,D) where $E_{1}\subseteq A\times B$, $E_{2}\subseteq B\times C$ and $E_{3}\subseteq C\times D$. Then, $R_{1}\circ \left(R_{2}\circ R_{3}\right)=\left(R_{1}\circ R_{2}\right)\circ R_{3}.$

**Proof.**  $\forall \left(a,b\right)\in E_{1}\subseteq A\times B$, $\left(b,c\right)\in E_{2}\subseteq B\times C$ and $\left(c,d\right)\in E_{3}\subseteq C\times D$, let $R_{1}\circ \left(R_{2}\circ R_{3}\right)\left(a,d\right)$ $=\left\{\langle \left(a,d\right),T_{R_{1}\circ \left(R_{2}\circ R_{3}\right)\left(a,d\right)}\left(u\right),I_{R_{1}\circ \left(R_{2}\circ R_{3}\right)\left(a,d\right)}\left(u\right),F_{R_{1}\circ \left(R_{2}\circ R_{3}\right)\left(a,d\right)}\left(u\right)\rangle :\left(a,d\right)\in A\times D,u\in U\right\}$ and $\left(R_{1}\circ R_{2}\right)\circ R_{3}\left(a,d\right)=\left\{\langle \left(a,d\right),T_{\left(R_{1}\circ R_{2}\right)\circ R_{3}\left(a,d\right)}\left(u\right),I_{\left(R_{1}\circ R_{2}\right)\circ R_{3}\left(a,d\right)}\left(u\right),F_{\left(R_{1}\circ R_{2}\right)\circ R_{3}\left(a,d\right)}\left(u\right)\rangle :\left(a,d\right)\in A\times D,u\in U\right\}.$

To prove the equality, we have to show that $T_{R_{1}\circ \left(R_{2}\circ R_{3}\right)\left(a,d\right)}\left(u\right)=T_{\left(R_{1}\circ R_{2}\right)\circ R_{3}\left(a,d\right)}\left(u\right),I_{R_{1}\circ \left(R_{2}\circ R_{3}\right)\left(a,d\right)}\left(u\right)=I_{\left(R_{1}\circ R_{2}\right)\circ R_{3}\left(a,d\right)}\left(u\right)$ and $F_{R_{1}\circ \left(R_{2}\circ R_{3}\right)\left(a,d\right)}\left(u\right)=F_{\left(R_{1}\circ R_{2}\right)\circ R_{3}\left(a,d\right)}\left(u\right)$. Therefore,

$$\begin{array}{cc} p_{{\left(R_{1}\circ R_{2}\right)}^{-1}\left(a,c\right)}\left(u\right) & =p_{\left(R_{1}\circ R_{2}\right)\left(c,a\right)}\left(u\right)\\ & =max\left(p_{R_{1}\left(c,b\right)}\left(u\right),p_{R_{2}\left(b,a\right)}\left(u\right)\right),=max\left(p_{R_{2}\left(b,a\right)}\left(u\right),p_{R_{1}\left(c,b\right)}\left(u\right)\right),\\ & =max[min\left(p_{G(b)}\left(u\right),p_{H(a)}\left(u\right)\right),min\left(p_{k(c)}\left(u\right),p_{G(b)}\left(u\right)\right)],\\ & =max[min\left(p_{H(a)}\left(u\right),p_{G(b)}\left(u\right)\right),min\left(p_{G(b)}\left(u\right)\right),p_{k(c)}\left(u\right)],\\ & =max(p_{R_{2}^{-1}\left(a,b\right)}\left(u\right),p_{R_{1}^{-1}\left(b,c\right)}\left(u\right)),\\ & =p_{R_{2}^{-1}\circ R_{1}^{-1}\left(a,c\right)}\left(u\right), \end{array}$$

which implies that $p_{R_{1}\circ \left(R_{2}\circ R_{3}\right)\left(a,d\right)}\left(u\right)=p_{\left(R_{1}\circ R_{2}\right)\circ R_{3}\left(a,d\right)}\left(u\right)$. Similarly, we can show that $\mu_{R_{1}\circ \left(R_{2}\circ R_{3}\right)\left(a,d\right)}\left(u\right)=\mu_{\left(R_{1}\circ R_{2}\right)\circ R_{3}\left(a,d\right)}\left(u\right)$, proving that $T_{R_{1}\circ \left(R_{2}\circ R_{3}\right)\left(a,d\right)}\left(u\right)=T_{\left(R_{1}\circ R_{2}\right)\circ R_{3}\left(a,d\right)}\left(u\right)$. ☐

The proofs for the identity and falsity terms follow similarly.

## 5. Equivalence Relations and Equivalence Classes on CNSESs

In this section, we define four types of CNSERs of reflexive, symmetric, transitive and equivalence relations. We also define the concept of equivalence class and give a proposition combining all of these concepts.

**Definition** **11.** *Let (R,C) be a CNSER on (H,A). Then,*

*1.*  (R,C) is said to be reflexive, if R(a,a)∈(R,C),∀a∈A.
*2.*  (R,C) is said to be symmetric, if R(a,b)∈(R,C)⇒R(b,a)∈(R,C),∀(a,b)∈C⊆A×A.
*3.*  (R,C) is said to be transitive, if ∀a,b,c∈A, R(a,b)∈(R,C) and R(b,c)∈(R,C)⇒R(a,c)∈(R,C).

Now, we propose the definition of equivalence CNSER along with an illustrative example, followed by the definition of equivalence class in CNSES.

**Definition** **12.** A CNSER (R,C) on a CNSES (H,A) is said to be equivalent if it is reflexive, symmetric and transitive.

**Example** **4.** Consider a CNSES (H,A) over a universe U, where $U=\{u_{1},u_{2}\}$ and $A=\{\left(a_{1},p,1\right),\left(a_{1},q,0\right),\left(a_{2},p,1\right)\}$, where X={p,q} is a set of experts.

*The CNSES (H,A) can be defined as follows:*

$$\begin{array}{cc} (H,A)= & \{\left(\left(a_{1},p,1\right),\left\{\frac{\langle 0.4e^{j0.9\Pi },\;0.6e^{j0.1\Pi },\;0.6e^{j0.1\Pi }\rangle }{u_{1}},\frac{\langle 0.8e^{j0.5\Pi },\;0.7e^{j0.3\Pi },\;0.8e^{j0.7\Pi }\rangle }{u_{2}}\right\}\right),\\ & \left(\left(a_{1},q,0\right),\left\{\frac{\langle 0.2e^{j0.5\Pi },\;0.7e^{j0.1\Pi },\;0.1e^{j0.3\Pi }\rangle }{u_{1}},\frac{\langle 0.5e^{j0.6\Pi },\;0.4e^{j0.6\Pi },\;0.7e^{j0.4\Pi }\rangle }{u_{2}}\right\}\right),\\ & \left(\left(a_{2},p,1\right),\left\{\frac{\langle 0.5e^{j0.3\Pi },\;0.5e^{j0.2\Pi },\;0.2e^{j0.2\Pi }\rangle }{u_{1}},\frac{\langle 0.8e^{j0.8\Pi },\;0.1e^{j0.4\Pi },\;0.7e^{j0.3\Pi }\rangle }{u_{2}}\right\}\right)\}. \end{array}$$

*Consider a CNSER (R,C) on (H,A) defined as:*

$$\begin{array}{cc} (R,C)= & \{\left(\left(\left(a_{1},p,1\right),\left(a_{1},p,1\right)\right),\left\{\frac{\langle 0.4e^{j0.9\Pi },\;0.6e^{j0.1\Pi },\;0.6e^{j0.1\Pi }\rangle }{u_{1}},\frac{\langle 0.8e^{j0.5\Pi },\;0.7e^{j0.3\Pi },\;0.8e^{j0.7\Pi }\rangle }{u_{2}}\right\}\right),\\ & \left(\left(\left(a_{1},q,0\right),\left(a_{1},q,0\right)\right),\left\{\frac{\langle 0.2e^{j0.5\Pi },\;0.7e^{j0.1\Pi },\;0.1e^{j0.3\Pi }\rangle }{u_{1}},\frac{\langle 0.5e^{j0.6\Pi },\;0.4e^{j0.6\Pi },\;0.7e^{j0.4\Pi }\rangle }{u_{2}}\right\}\right),\\ & \left(\left(\left(a_{1},p,1\right),\left(a_{2},p,1\right)\right),\left\{\frac{\langle 0.4e^{j0.3\Pi },\;0.6e^{j0.2\Pi },\;0.6e^{j0.2\Pi }\rangle }{u_{1}},\frac{\langle 0.8e^{j0.5\Pi },\;0.7e^{j0.4\Pi },\;0.8e^{j0.7\Pi }\rangle }{u_{2}}\right\}\right),\\ & \left(\left(\left(a_{2},p,1\right),\left(a_{1},p,1\right)\right),\left\{\frac{\langle 0.4e^{j0.3\Pi },\;0.6e^{j0.2\Pi },\;0.6e^{j0.2\Pi }\rangle }{u_{1}},\frac{\langle 0.8e^{j0.5\Pi },\;0.7e^{j0.4\Pi },\;0.8e^{j0.7\Pi }\rangle }{u_{2}}\right\}\right),\\ & \left(\left(\left(a_{2},p,1\right),\left(a_{2},p,1\right)\right),\left\{\frac{\langle 0.5e^{j0.3\Pi },\;0.5e^{j0.2\Pi },\;0.2e^{j0.2\Pi }\rangle }{u_{1}},\frac{\langle 0.8e^{j0.8\Pi },\;0.1e^{j0.4\Pi },\;0.7e^{j0.3\Pi }\rangle }{u_{2}}\right\}\right)\}. \end{array}$$

The CNSER (R,C) is shown to be reflexive, symmetric and transitive. Hence, it is an equivalence relation.

**Definition** **13.** *If (H,A) is a CNSES, then the equivalence class of H(a) is defined as:*

[H(a)]={H(b):H(b)RH(a),∀a,b∈A}.

**Example** **5.** *Consider Example 4; we have:*

$$\left[H\left(\left(a_{1},p,1\right)\right)\right]=\left\{H\left(\left(a_{1},p,1\right)\right),H\left(\left(a_{2},p,1\right)\right)\right\}=\left[H\left(\left(a_{2},p,1\right)\right)\right].$$

This means that the approximation $H(\left(a_{1},p,1\right))$ is related to two approximations $H(\left(a_{1},p,1\right))$ and $H(\left(a_{2},p,1\right))$. These two approximations are also related to $H(\left(a_{2},p,1\right))$.

**Proposition** **2.** If (R,C) is an equivalence relation on a CNSES (H,A), then for any H(a) and H(b)∈(H,A), we have H(a)RH(b), if and only if [H(a)]=[H(b)].

**Proof.**  (⇒) Suppose H(a)RH(b). Let H(α)∈[H(a)]. Then, H(α)RH(a), and since (R,C) is transitive, it follows that H(α)RH(b). Hence, H(α)∈[H(b)] and, thus, [H(a)]⊆[H(b)]. In the same manner, [H(b)]⊆[H(a)]. Therefore, [H(a)]=[H(b)].

(⇐) Suppose [H(a)]=[H(b)]. Since (R,C) is reflexive, then H(a)RH(a). Hence, H(a)∈[H(a)]=[H(b)] and, thus, H(a)RH(b). Therefore, H(a)RH(b), if and only if [H(a)]=[H(b)]. ☐

## 6. Conclusions

We have established a novel structure for the relation between two complex neutrosophic soft expert sets, called the complex neutrosophic soft expert relation. We defined the concept of the complex neutrosophic soft expert set and the Cartesian product of two complex neutrosophic soft expert sets as a prerequisite to define the CNSER. A real-life problem was discussed and analyzed, using the key features of CNSER. Then, a generalized algorithm is introduced and applied to the CNSER model to solve an MADM problem. A comparison of the current models to the CNSER was presented, and the preferability of CNSER was revealed. We then presented the inverse and complement of CNSER and the composition of CNSERs and derived some properties of CNSER along with illustrative examples. Finally, the concepts of equivalence relations and equivalence classes of CNSESs were defined. CNSER seems to be a promising new concept, paving the way toward numerous possibilities for future research. A neutrosophic cognitive map is a neutrosophic directed graph that depicts the causal interaction between the various parameters. Introducing CNSER to neutrosophic cognitive map applications may provide a powerful tool for describing the time of the interaction between the neutrosophic cognitive map parameters where the knowledge of experts is contemplated. The structure of the CNSER is also rehabilitated to describe the relations between periodic phenomena that have uncertain data where the amplitude terms represent the uncertainty and the phase terms represent the periodicity semantic. Thus, the CNSER may provide a powerful framework to represent problems with uncertainty and periodicity simultaneously in the field of physics, signal processing, stock marketing, and so on. The CNSER may also be used as a new descriptor for the relation between vector quantities, where the amplitude and phase terms of the CNSER, respectively, represent the magnitudes and the directions of the vector quantities.

## Figures and Tables

**Table 1:** Values of $\left(\hat{R},C\right)$ and $Z_{\hat{R}\left(\alpha ,\beta \right).}\left(u\right)$.

*U*	$u_{1}$	$u_{2}$	$u_{3}$
$\left(\left(e_{1},p,1\right),\left({\overset{´}{e}}_{1},p,1\right)\right)$	〈0.014,0.153,0.94〉	〈0.907,0.313,0.06〉	〈0.024,0.128,0.787〉
	0.307	0.845	0.37
$\left(\left(e_{1},q,1\right),\left({\overset{´}{e}}_{1},q,1\right)\right)$	〈0.012,0.137,0.847〉	〈0.753,0.28,0.128〉	〈0.047,0.273,0.753〉
	0.343	0.782	0.340
$\left(\left(e_{1},p,0\right),\left({\overset{´}{e}}_{1},p,0\right)\right)$	〈0.787,0.923,0.353〉	〈0.03,0.907,0.94〉	〈0.433,0.932,0.4〉
	0.504	0.061	0.367
$\left(\left(e_{1},q,0\right),\left({\overset{´}{e}}_{1},q,0\right)\right)$	〈0.727,0.923,0.327〉	〈0.075,0.907,0.932〉	〈0.46,0.923,0.307〉
	0.492	0.079	0.41
$\left(\left(e_{2},p,1\right),\left({\overset{´}{e}}_{2},p,1\right)\right)$	〈0.03,0.068,0.753〉	〈0.84,0.247,0.056〉	〈0.503,0.94,0.327〉
	0.403	0.846	0.412
$\left(\left(e_{2},q,1\right),\left({\overset{´}{e}}_{2},q,1\right)\right)$	〈0.020,0.037,0.88〉	〈0.813,0.247,0.065〉	〈0.036,0.22,0.907〉
	0.368	0.834	0.303
$\left(\left(e_{2},p,0\right),\left({\overset{´}{e}}_{2},p,0\right)\right)$	〈0.7,0.94,0.22〉	〈0.115,0.961,0.992〉	〈0.503,0.94,0.327〉
	0.513	0.054	0.412
$\left(\left(e_{2},q,0\right),\left({\overset{´}{e}}_{2},q,0\right)\right)$	〈0.623,0.94,0.22〉	〈0.087,0.915,0.932〉	〈0.46,0.932,0.36〉
	0.488	0.08	0.389
$\left(\left(e_{3},p,1\right),\left({\overset{´}{e}}_{3},p,1\right)\right)$	〈0.873,0.06,0.213〉	〈0.006,0.093,0.72〉	〈0.03,0.307,0.873〉
	0.867	0.398	0.283
$\left(\left(e_{3},q,1\right),\left({\overset{´}{e}}_{3},q,1\right)\right)$	〈0.813,0.128,0.253〉	〈0.024,0.22,0.693〉	〈0.012,0.4,0.847〉
	0.811	0.370	0.255
$\left(\left(e_{3},p,0\right),\left({\overset{´}{e}}_{3},p,0\right)\right)$	〈0.045,0.988,1〉	〈0.46,0.929,0.527〉	〈0.727,0.907,0.407〉
	0.019	0.335	0.471
$\left(\left(e_{3},q,0\right),\left({\overset{´}{e}}_{3},q,0\right)\right)$	〈0.051,0.94,0.88〉	〈0.467,0.89,0.433〉	〈0.6,0.847,0.433〉
	0.077	0.381	0.44
$\left(\left(e_{4},p,1\right),\left({\overset{´}{e}}_{4},p,1\right)\right)$	〈0.093,0.587,0.94〉	〈0.036,0.533,0.94〉	〈1,0.16,0.068〉
	0.189	0.188	0.924
$\left(\left(e_{4},q,1\right),\left({\overset{´}{e}}_{4},q,1\right)\right)$	〈0.22,0.593,0.667〉	〈0.06,0.507,0.907〉	〈0.907,0.253,0.145〉
	0.32	0.215	0.836
$\left(\left(e_{4},p,0\right),\left({\overset{´}{e}}_{4},p,0\right)\right)$	〈0.293,0.567,0.22〉	〈0.88,0.62,0.153〉	〈0.054,0.955,0.992〉
	0.502	0.702	0.036
$\left(\left(e_{4},q,0\right),\left({\overset{´}{e}}_{4},q,0\right)\right)$	〈0.32,0.72,0.347〉	〈0.82,0.587,0.247〉	〈0.069,0.882,0.923〉
	0.418	0.662	0.088

**Table 2:** Numerical grade for agree-SVNSES.

*U*	$u_{i}$	Highest Numerical Grade
$\left(\left(e_{1},p,1\right),\left({\overset{´}{e}}_{1},p,1\right)\right)$	$u_{2}$	0.845
$\left(\left(e_{1},q,1\right),\left({\overset{´}{e}}_{1},q,1\right)\right)$	$u_{2}$	0.782

**Table 3:** Numerical grade for disagree-single-valued neutrosophic soft expert set (SVNSES).

*U*	$u_{i}$	Highest Numerical Grade
$\left(\left(e_{1},p,0\right),\left({\overset{´}{e}}_{1},p,0\right)\right)$	$u_{1}$	0.504
$\left(\left(e_{1},q,0\right),\left({\overset{´}{e}}_{1},q,0\right)\right)$	$u_{1}$	0.492

**Table 4:** The score $r_{i}=K_{i}-S_{i}$.

*i*	$K_{i}$	$S_{i}$	$r_{i}$
1	0	0.966	−0.966
2	1.627	0	1.627
3	0	0	0
